# Supplementary material for: Creating fluorescent quantum defects in carbon nanotubes using hypochlorite and light
Source: Nat Commun. 2019 Jun 28;10:2874. doi: 10.1038/s41467-019-10917-3 (PMC6599008; doi:10.1038/s41467-019-10917-3)
Supplement: Supplementary file 1 — Supplementary Information [file 41467_2019_10917_MOESM1_ESM.pdf]

Supplementary Information

Creating fluorescent quantum defects in carbon nanotubes using hypochlorite and light

Lin et al.

Supplementary Methods

Preparation of (6,5)-enriched SWCNTs

We purified the CoMoCAT SWCNTs using a modified version of the gel chromatography method described in prior publications[1, 2]. The CoMoCAT SWCNTs were dispersed in 50 mL of 1% aqueous SC. A 50 mL portion of 1% SDS was then mixed with the dispersed SWCNTs to give a stock suspension containing 0.5% SC and 0.5% SDS. Then SDC solution was added to give a final surfactant concentration of 0.5% SC + 0.5% SDS + 0.035% SDC. This sample was loaded onto a column packed with S-200 Sephacryl gel. The eluent was collected and then diluted with a solution of 0.5% SC and 0.5% SDS to give a final surfactant concentration of 0.5% SC + 0.5% SDS + 0.023% SDC. The adsorbed SWCNTs remaining on the gel were larger diameter species. The eluent was added to a larger gel column for (6,5) adsorption. The column was washed with 0.5% SC + 0.5% SDS + 0.023% SDC and then the SWCNTs were eluted with 0.5% SC + 0.5% SDS + 0.035% SDC solution. This SWCNT suspension was finally washed with 1% SC and then concentrated using tangential flow filtration.

Oxygen doping protocol for small volume samples

1. Dilute the stock solution (SWCNTs dispersed in 1% SC) with DI water and add the NaClO stock (~150 mM) to prepare the SWCNT solution for reaction at desired SC and NaClO concentration.
2. Fully irradiate the sample with 300 nm light while monitoring the E_{11}^{*} emission intensity. Stop when E_{11}^{*} emission reaches a maximum. (Make sure the whole sample is illuminated to give the best results). The reaction time is around 40 - 60 s with ~29 mW cm^{-2} irradiation intensity.
3. Add extra SDC or SC (10%) to the reacted solution to reach 0.2 - 1% of final surfactant concentration.

4. (Optional) Place the reacted solution in a dialysis tube and concentrate the solution (in 0.2% SC) using water absorbent (Spectra/Gel®). Concentration by a factor of 10 is ideal because the concentration of surfactant reaches 2%.
5. (Optional) If higher concentration is needed, use tangential flow filtration to concentrate the SWCNTs and keep the surfactant concentration around 1 - 2%.

Note: Higher stock SWCNT concentration makes the doping procedure easier because of the following reasons: (1) The higher SWCNT concentration under the same SC concentration leads to more exposed SWCNT surfaces. The reaction proceeds faster when the surface coating is incomplete. (2) Higher SWCNT concentration means more SWCNT products. (3) The resulting SWCNT concentration can reach OD ~3 per cm without further concentration steps. (4) Similar amounts of NaClO are required for reactions under low and high SWCNT concentrations.

Protocol for finding the optimum doping condition

1. Dilute the SWCNT solution so that the concentration of SC is less then the CMC, usually around 0.035 - 0.07%. Larger diameter SWCNTs need lower concentration of SC because SC more effectively coats larger diameter SWCNTs.
2. Add ~1 mM NaClO into solution. Several conditions need to be tested in order to find the optimum NaClO concentration. For (6,5)-SWCNTs, Fig. 3c is a good reference. For unsorted CoMoCAT samples, the SWCNT fluorescence tends to be quenched when the NaClO concentration is around 0.7 mM or slightly lower. A concentration slightly higher than the minimum NaClO concentration that will not quench the SWCNTs is the best.
3. Find the conditions for the highest shifted emission intensity again by checking several SC concentrations around the value used in step 1 with the NaClO concentration used in step 2.
4. Repeat step 2 to optimize the conditions.

Supplementary Discussion

Optical properties of the SWCNT stock solutions. We mainly used CoMoCAT and (6,5) SWCNTs for this study. The absorbance spectra in Supplementary Fig. 1a and Supplementary Fig. 1b were measured from diluted stock solutions and multiplied by the dilution factor. The (6,5)-SWCNT samples contain trace amounts of (9,1) but the purity is estimated at more than 90% based on the literature. Gel purification removed most of the impurities in the CoMoCAT sample. The comparable D/G ratios suggest that the nonfluorescent defect densities of both samples are very similar. We also observed higher 2D peaks in CoMoCAT samples, possibly because of graphene impurities (Supplementary Fig. 1c,d).

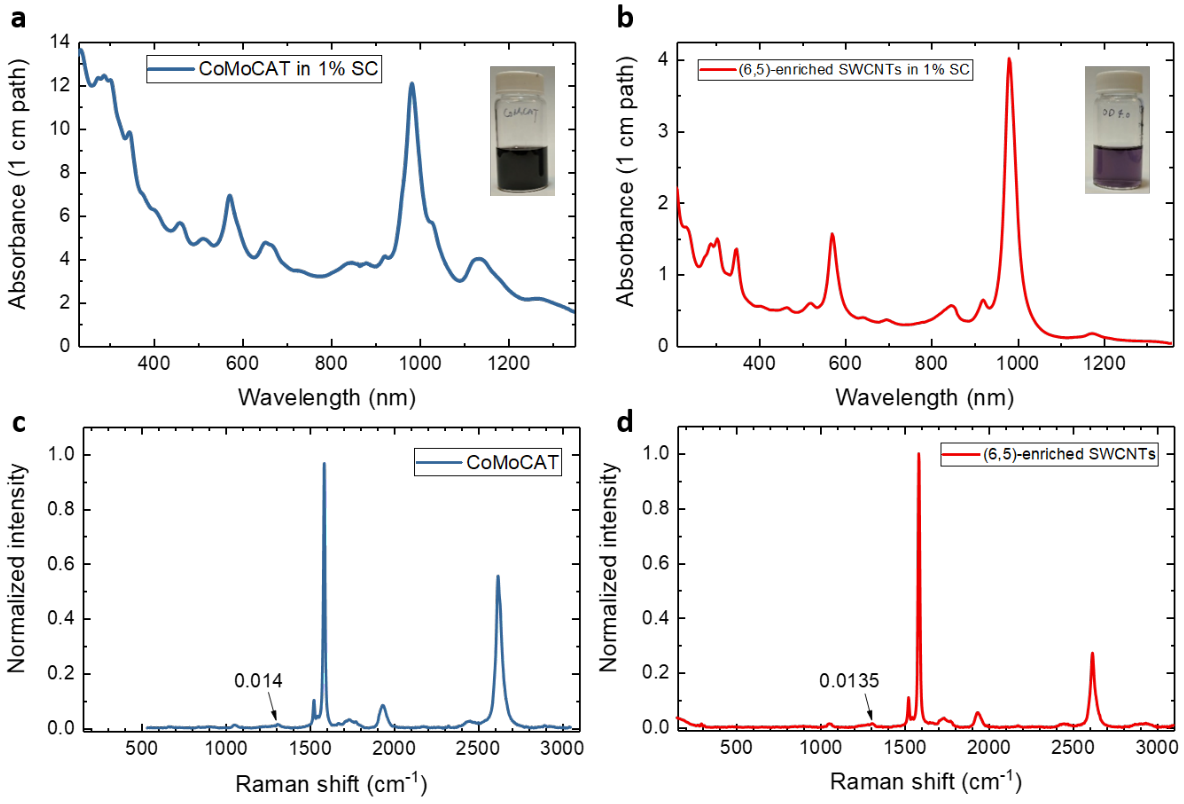

Supplementary Fig. 1 Optical properties of (6,5) and CoMoCAT SWCNTs. **a**, Absorption spectrum of the CoMoCAT SWCNTs in 1% SC. **b**, Absorption spectrum of (6,5)-SWCNTs in 1% SC. **c**, Raman spectrum of the CoMoCAT SWCNTs. **d**, Raman spectrum of the (6,5)-SWCNTs.

Emission spectra in frequency units. The emission spectra in Supplementary Fig. 2 have been converted to show wavenumbers on the x-axis and quanta on the y-axis. The area ratio of O-doped to pristine SWCNTs shows the quantum yield ratio, which is 2.6 in this case. The actual increase of the quantum yield should be slightly higher than the apparent value because water absorbs light at longer wavelengths. The amount of increase is also strongly related to the initial condition of the pristine nanotubes, such as defect density and lengths. Lower density of non-fluorescent defects on pristine SWCNTs and longer SWCNT lengths could raise the quantum yield of the pristine nanotubes, and thus, decrease the quantum yield ratio, $\phi_{\text{O-doped}}/\phi_{\text{pristine}}$. The FWHM is 318 cm^{-1} for the E_{11} peak and 436 cm^{-1} for the E_{11}^{*} peak. Supplementary figure 2b shows normalized and aligned spectra with the frequency zero set to the E_{11} peak for pristine SWCNTs and to the E_{11}^{*} peak for O-doped SWCNTs. The low frequency side bands for E_{11} and E_{11}^{*} can be seen to lie at similar positions (~1141 cm^{-1} lower than the main peaks) with similar intensities. This sideband in pristine nanotubes has been assigned to X_1 band, which is the emission from the dark K-momentum exciton. We suspect the low frequency sideband in the treated sample might arise from the same source and therefore could be assigned to X_1^{*}. Also, this peak appears different from the assigned parallel epoxide emission $E_{11}^{*,-}$, which should be near 7500 cm^{-1} (at 1333 nm or 1411 cm^{-1} lower than E_{11}^{*}). However, a minor contribution from $E_{11}^{*,-}$ emission cannot be excluded (see also page 22), and the accurate assignments of the sidebands need further study.

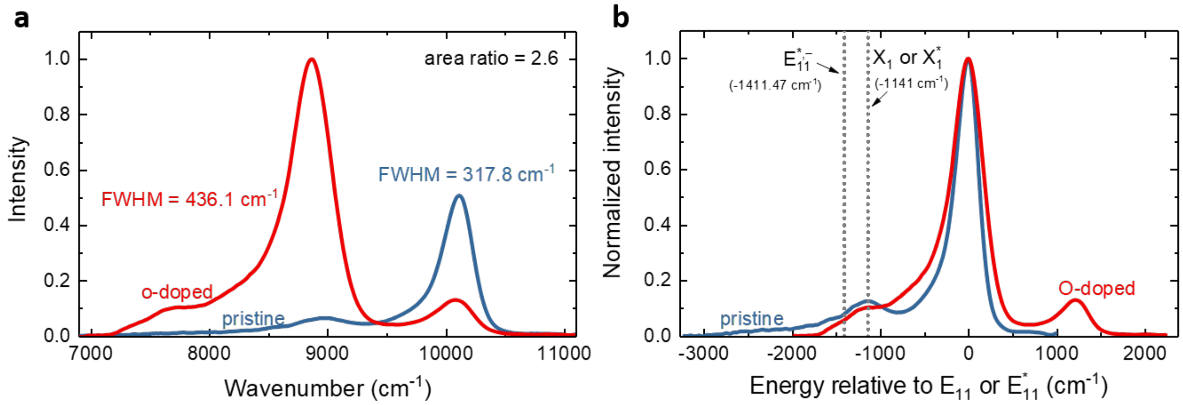

Supplementary Fig. 2 Emission spectra of pristine and O-doped (6,5)-enriched SWCNTs converted to energy scale. **a**, The area ratio is ca. 2.6. The FWHM is broadened from 317.8 to 436.1 cm^{-1}. **b**, The spectral position and shapes of both pristine and O-doped SWCNTs are very similar, including the sideband position and intensity.

Absorption of the E_{11}^{*} band. Supplementary Fig. 3 plots the difference absorbance spectrum between O-doped and pristine samples. We believe that the peak shows the weak absorption arising from the O-doped sites. This feature has a peak wavelength of ~1114 nm and a FWHM of ~54 nm. The defect density is so low that this absorption peak is very hard to measure. The absorption coefficient might be extracted if the defect density can be quantified. A future determination of this absorption coefficient would allow accurate measurements of doping density.

It is of fundamental interest to understand the vibrational reorganization energy for E_{11}^{*} transitions. As shown in Supplementary Fig. 3b, the relative energies can be written as follows assuming vertical (Franck-Condon) transitions:

$$E_{11}^{*,abs} = \lambda_{X^-} + E_{11}^{*,em} + \lambda_G \quad (1)$$

or

$$E_{11}^{*,abs} - E_{11}^{*,em} = \lambda_{X^-} + \lambda_G \,. \quad (2)$$

Therefore, the energy difference between absorption and emission equals the total reorganization energy, which is $\lambda_{total} = \lambda_{X^-} + \lambda_G$. The λ_{total} obtained from this work is ~11.9 meV, which is much smaller than the reported calculated λ_G of 70 meV[3]. Dense oxygen doping in our treated sample might result in a reduced reorganization energy, which is also observed in the *sp*[3] doped samples[3].

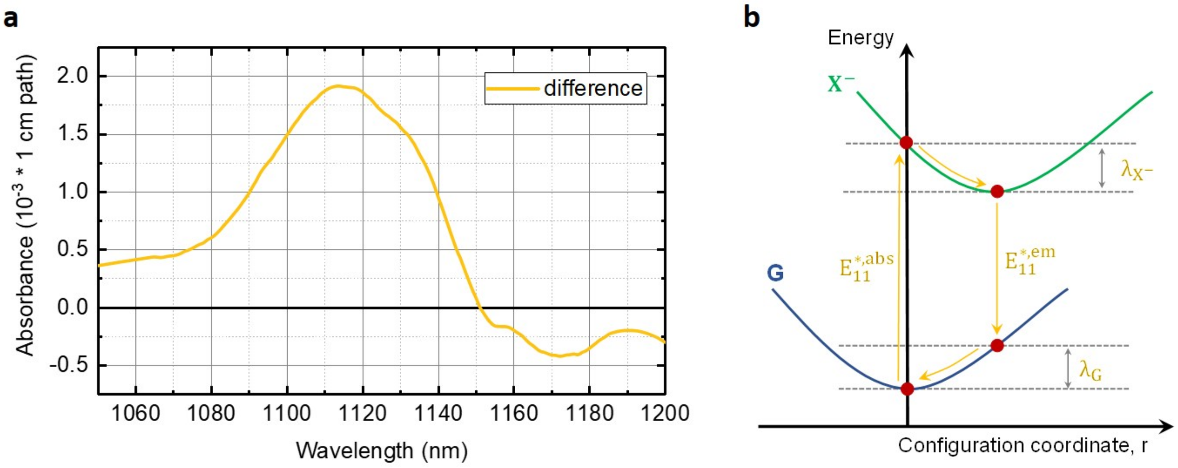

Supplementary Fig. 3 E_{11}^{*} absorption and energy diagram at defect site. **a**, Difference of absorption spectra between treated and pristine samples. **b**, Energy diagram of an ether-SWCNT.

Up-conversion of pristine and O-doped SWCNTs. SWCNT up-conversion was first reported by Akizuki *et al.*[4] in 2015. Light with photon energy lower than E_{11} can still excite the SWCNTs in a thermally assisted process. The E_{11} emission intensity of pristine SWCNTs excited at 1125 nm is ca. 9.35% compared to excitation at 565 nm, which matches previous observations[4, 5]. The E_{11} intensity from the up-conversion excitation for O-doped SWCNTs is ~2.67 times lower than that for the pristine SWCNTs (0.0235/0.0627=2.67 from Supplementary Fig. 4b). This ratio is not too far away from the E_{11} intensity ratio of pristine to O-doped SWCNTs excited at 565 nm, which is around 3.29 (from Supplementary Fig. 4a). The lowered E_{11} ratio from the up-conversion transition might indicate a larger absorption cross-section at the O-doped site compared to the thermal assisted absorption of the pristine structure. However, our distance between O-doped sites should be much smaller than the exciton diffusion length, which is around 200 nm. The escaped excitons from the traps are likely to re-enter a trapping site, lowering up-conversion efficiency. Lighter O-doping might help to produce higher up-conversion through defect-assisted exciton generation.

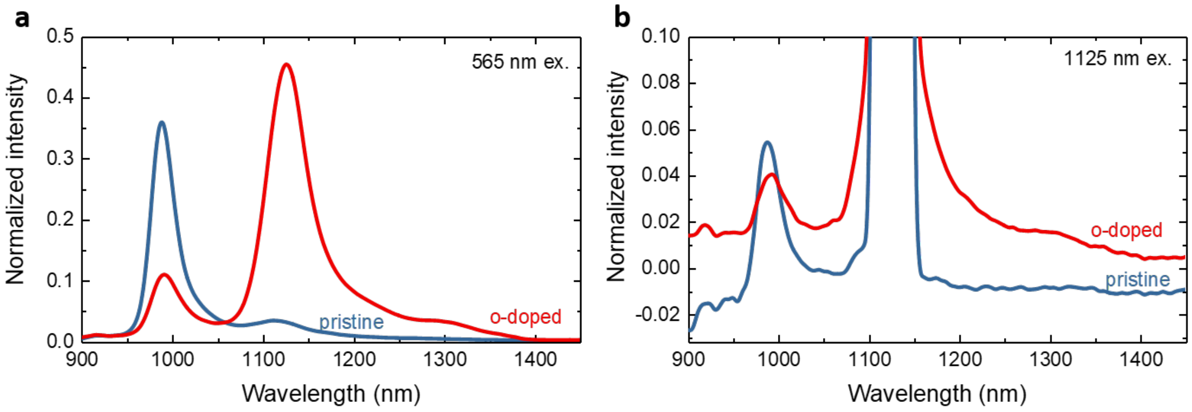

Supplementary Fig. 4 Emission spectra of pristine and O-doped (6,5)-SWCNTs excited at 565 (E_{11}) and 1125 (E_{11}^{*}) nm. **a**, Excitation at 565 nm. **b**, Excitation at 1125 nm.

Raman radial breathing mode. The Raman RBM peaks did not show a significant difference between pristine and O-doped samples. These three labeled peaks have been assigned in previous literature reports[6, 7].

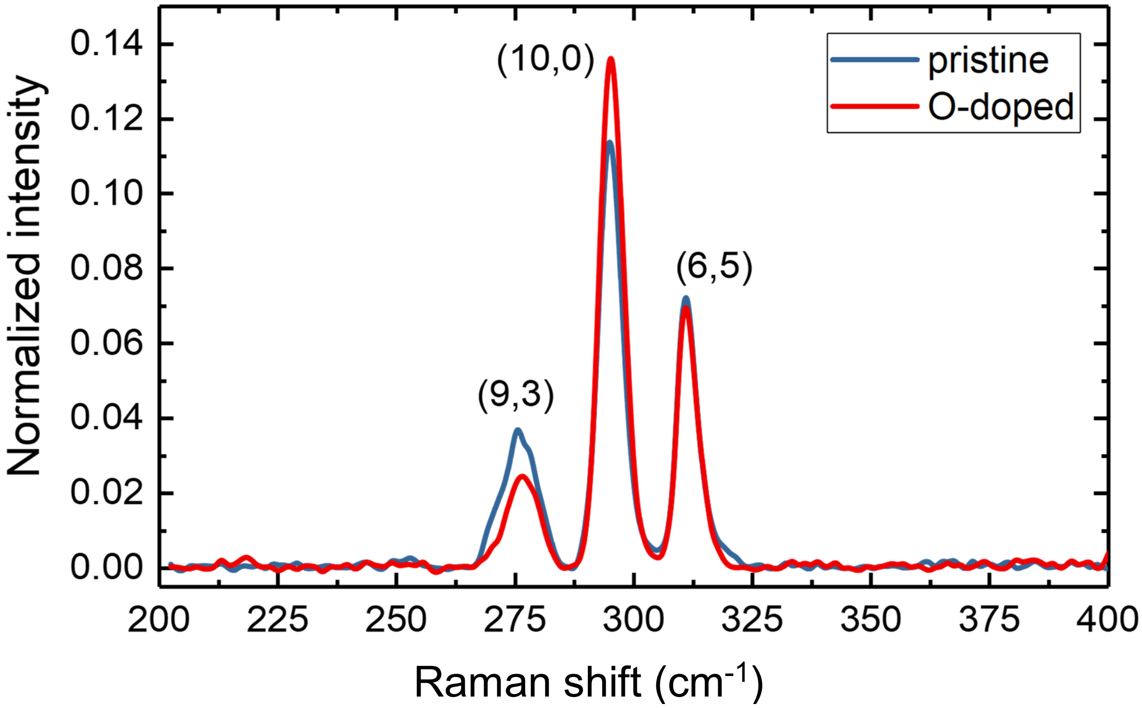

Supplementary Fig. 5 RBM spectra of pristine and O-doped (6,5)-enriched SWCNTs excited at 532 nm.

Optical properties of pristine and O-doped CoMoCAT SWCNTs. The results with CoMoCAT SWCNTs are very similar to those from the (6,5)-SWCNT samples. The near-armchair species seem to be less reactive than other species. In Supplementary Fig. 6c, the $E_{11}^{(8,3)}$ and $E_{11}^{(7,5)}$ emissions are obvious but in Supplementary Fig. 6d, the $E_{11}^{*,(8,3)}$ and $E_{11}^{*,(7,5)}$ peaks are hidden in the $E_{11}^{*,(6,5)}$ emission.

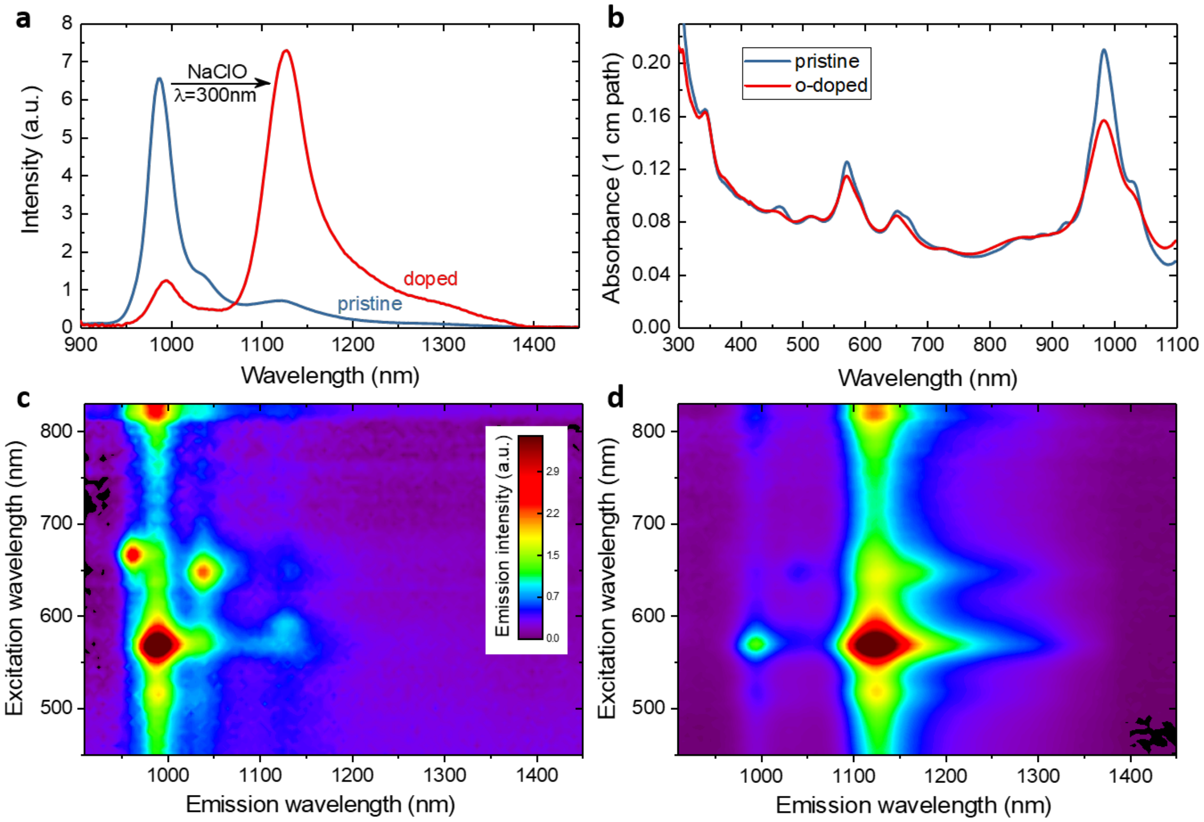

Supplementary Fig. 6 Optical properties of pristine and O-doped CoMoCAT SWCNTs. **a**, The emission spectra excited at 565 nm. **b**, The absorption spectra. **c**, The excitation-emission profile of the pristine CoMoCAT SWCNTs, showing (6,5), (8,3) and (7,5). **d**, The O-doped sample.

Oxygen doping of species other than (6,5). The oxygen doping reaction also proceeds for several species other than (6,5). Here, we doped oxygen into partially sorted HiPco SWCNTs. Supplementary Fig. 7a shows the excitation-emission profile of the pristine SWCNTs. The dominant species were (8,3), (6,5), (7,5), (10,2), (9,4), (7,6), and (8,4). Supplementary Fig. 7b shows the excitation-emission profile of the O-doped SWCNTs. The $E_{11}^{(8,3)}$ emission disappeared, indicating successful oxygen doping. The $E_{11}^{*,(8,3)}$ emission might be hidden by the dominant emissions from $E_{11}^{(7,6)}$ and $E_{11}^{*,(7,5)}$. The $E_{11}^{*,(6,5)}$ is obvious at 1126 nm and slightly overlaps with $E_{11}^{(8,4)}$. The (10,2) species seems less reactive under these conditions. The $E_{11}^{*,(8,4)}$ emission is observed at 1258 nm, and $E_{11}^{*,(7,6)}$ is at 1266 nm. Interestingly, the unshifted emissions of $E_{11}^{(9,4)}$ and $E_{11}^{(8,6)}$ become stronger after NaClO treatment. The $E_{11}^{*,(9,4)}$ and $E_{11}^{*,(8,6)}$ emissions were also not found in prior reports. Instead, the fluorescence recovery of the oxidized (9,4) and (8,6) was observed, perhaps because the oxidizing agents removed the nonfluorescent defects[8]. However, further study is still needed to clarify this special chirality-specific mechanism. The optimal concentration of SC for reaction is chirality dependent, as larger diameter SWCNTs require lower SC concentrations for the reaction to take place. The oxygen doping reaction was run under 0.03% SC in this case.

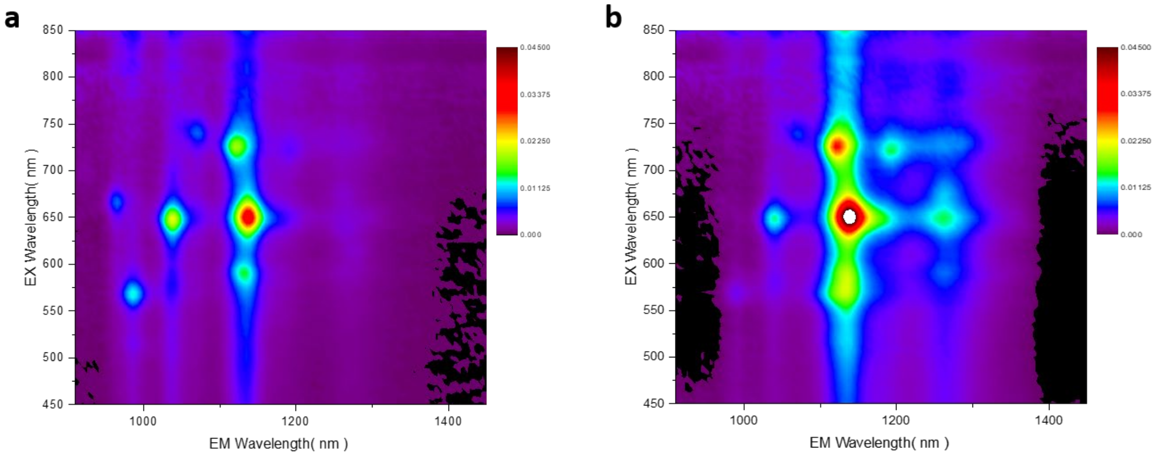

Supplementary Fig. 7 Excitation-emission map of pristine and O-doped sorted HiPco SWCNTs. **a**, Pristine sorted SWCNTs. **b**, O-doped sorted SWCNTs.

Photodissociation of ClO^{-} ions. ClO^{-} ions undergo photodissociation when illuminated with ~300 nm light. The absorbance of ClO^{-} is seen to decrease as the sample is irradiated at 300 nm. Here, we observe that most of the ClO^{-} ions have decomposed within 40 s, which matches the optimal illumination time for the reaction. We performed the O-doping reactions with several ClO^{-} concentrations in order to find conditions that maximized the E_{11}^{*} emission.

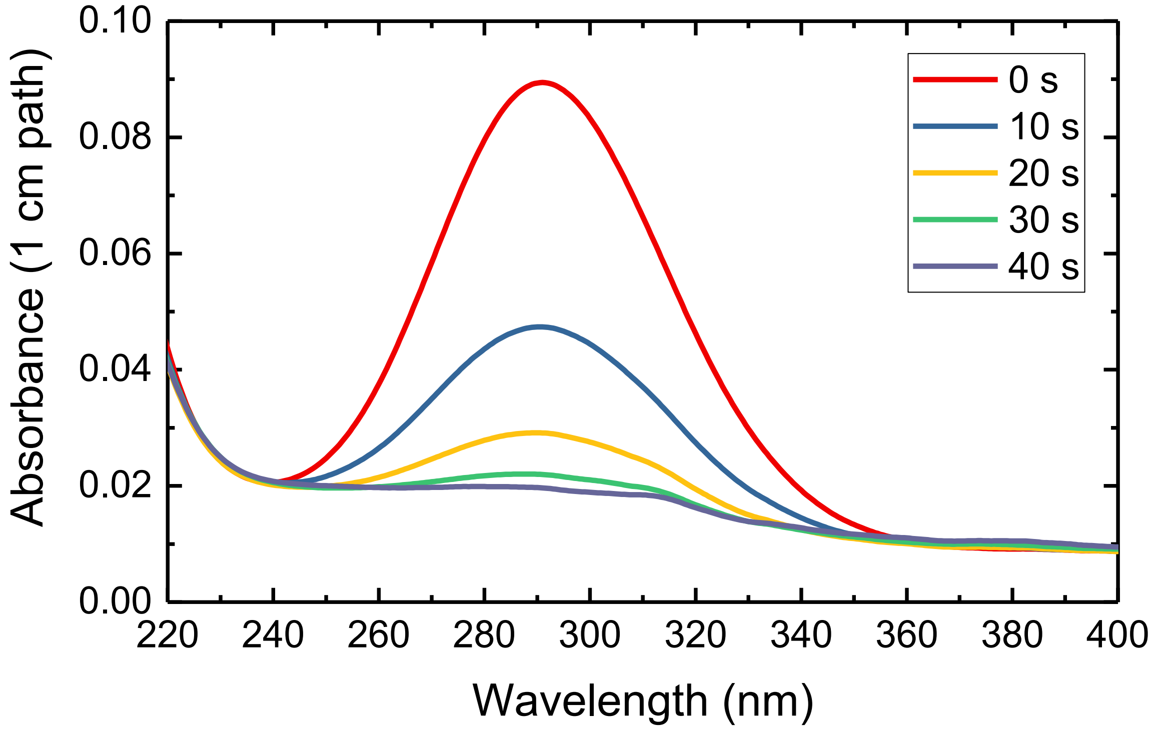

Supplementary Fig. 8 Absorption spectra of NaClO before and after illumination at 300 nm.

Sample stability. Here, we examined the stability of SWCNT samples in the presence of NaClO for 24 h. Supplementary Fig. 9a shows that the Raman D/G ratio increases only 10%, which is within the uncertainty of this measurement. This finding means that the NaClO did not severely alter the pristine structure. But the possibility of shortening of SWCNTs by NaClO cannot be excluded. Chiu *et al.*[8] have shown that although a low concentration of ClO^- ions does not affect the D/G ratio, the nanotube absorbance decreases. Higher concentrations of ClO^- ions can oxidize the SWCNT completely. Also, previous research[9] has found that oxidized graphene sheets degrade more easily than oxidized SWCNTs. Supplementary Fig. 9b and S9c both show decreased intensity of resonant spectral features after 24 h. The lowered emission intensities might result from aggregated or shortened SWCNTs. Supplementary Fig. 9b shows that the emission red-shifted slightly from 988 nm to 992 nm, indicating a possible environmental change around the SWCNT wall. The slightly broader emission also suggests possible aggregation during 24 h incubation. The lower absorption background in Supplementary Fig. 9c (or normalized in Supplementary Fig. 9d) might indicate that carbon related structures including amorphous carbon and small graphene sheets can be slowly decomposed by ClO^- ions. Also, the attack of the ClO^- ions might occur at non-fluorescent defect sites and therefore cause a decrease in pristine structure and shortened nanotube lengths. The overall density of non-fluorescent defects on nanotube walls might thereby be reduced.

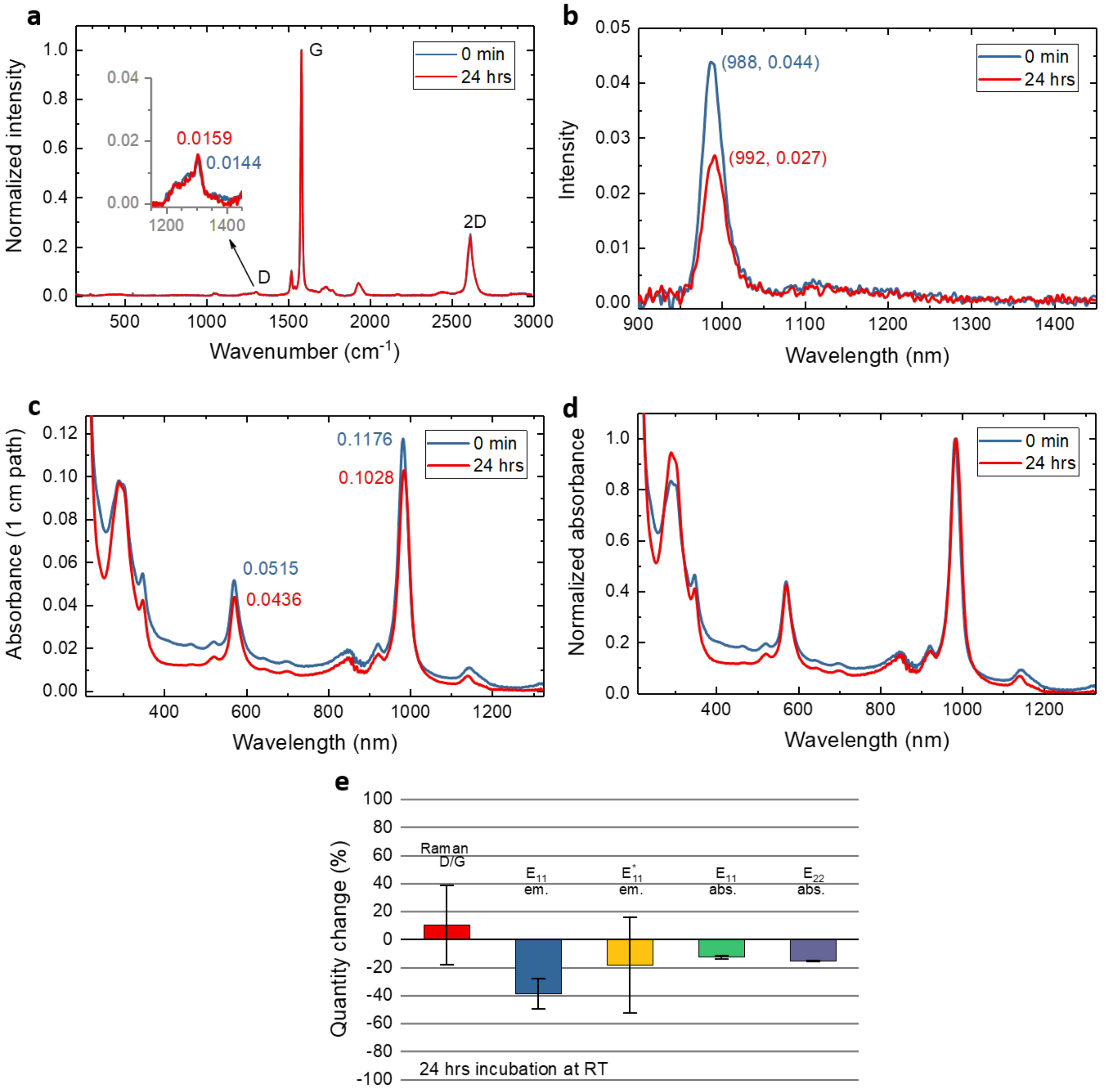

Supplementary Fig. 9 Sample stability in dark for 24 h. **a**, Raman spectra, **b**, Emission spectra, and **c**, Absorption spectra of (6,5)-enriched SWCNTs at time zero and time 24 h. **d**, Absorption spectra normalized to E_{11} peaks. **e**, Percentage change of O-doped to pristine SWCNT quantities in (**a**-**d**). Error bars are s.d. from spectral measurement noise.

300 nm illumination without NaClO. A sample of (6,5)-SWCNTs in 0.07% SC was illuminated by 300 nm light for 50 s while the solution was saturated with argon to prevent oxygen doping side effects. Supplementary Fig. 10a shows that the E_{11} fluorescence dropped by 83% after illumination and then recovered to 76% of initial value after 40 mins. This suggests that there is some largely reversible charge transfer reaction happening under 300-nm illumination. This charge transfer reaction creates some defects that induce small new sidebands that are not directly related to the fluorescent quantum defects. Supplementary Fig. 10c shows slightly lower and broader absorption at E_{11}. But in Supplementary Fig. 10d, the Raman spectrum shows little change in the low D/G ratio, suggesting no severe modification of the pristine structure.

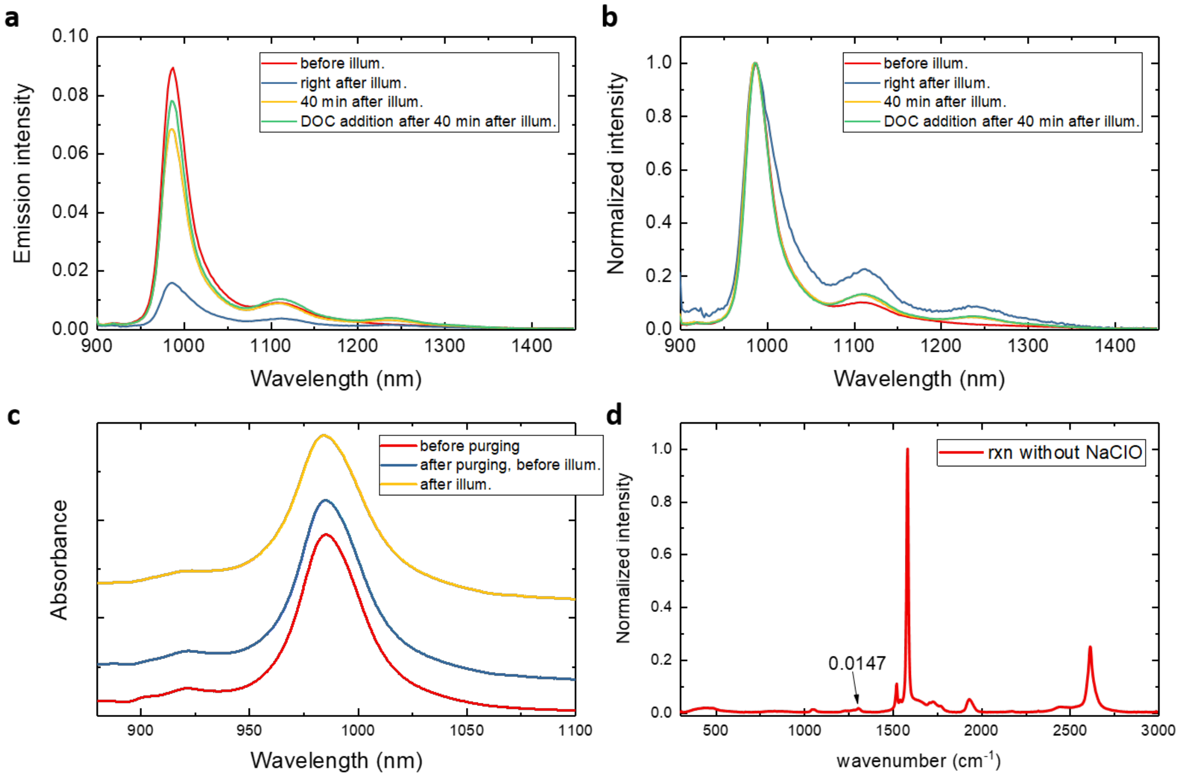

Supplementary Fig. 10 Characterization of NaClO-free (6,5)-SWCNTs illuminated by 300 nm light. **a**, Emission spectra of NaClO-free (6,5)-SWCNTs illuminated with 300 nm light. **b**, Normalized emission intensity of (**a**). **c**, Absorption spectra of E_{11}. **d**, Raman spectra of NaClO-free (6,5)-SWCNTs after 300-nm illumination.

NaClO control. Here, we illuminated the sample in the absence of ClO^- ions to check if dissolved oxygen molecules play any role in the doping mechanism. Supplementary Fig. 11a shows that the doping reaction did proceed very mildly under these conditions with short wavelength irradiation. The ratios of doping extent shown in Supplementary Fig. 11b reveal a clear threshold near 325 nm. This is consistent with a reaction channel involving ^{1}D oxygen doping, because ^{1}D oxygen atoms are generated only at wavelengths shorter than 320 nm.

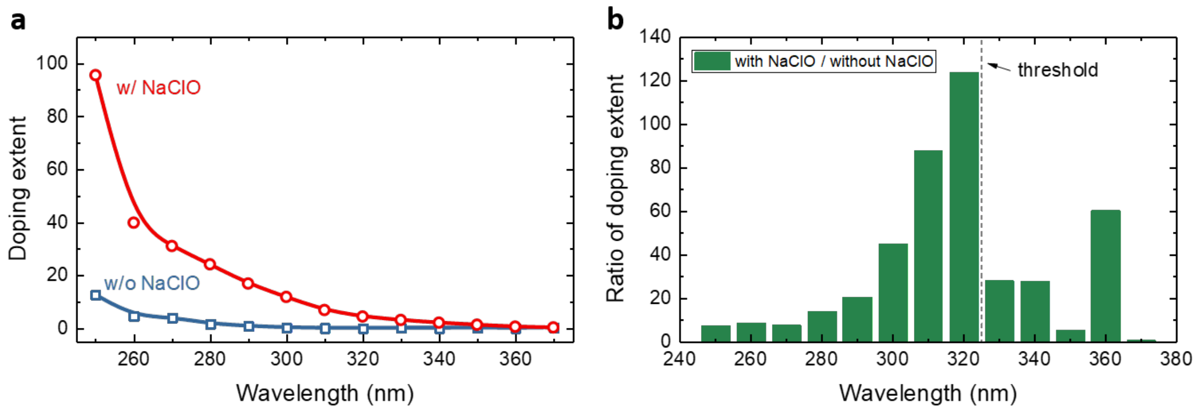

Supplementary Fig. 11 Doping extent with and without NaClO. **a**, The doping extent as a function of illumination wavelengths. **b**, The ratios of doping extent.

Generation of ^{1}D oxygen atoms. Prior studies have shown that ^{1}D oxygen atoms are generated upon photodissociation of hypochlorite ions at wavelengths shorter than ~320 nm. The rate of ^{1}D oxygen atom generation given a certain excitation wavelength can be estimated by the following equation:

$$\mathrm{O}(\,^1\mathrm{D})\ \text{photogeneration rate} = \frac{\#\ \text{of}\ \mathrm{O}(\,^1\mathrm{D})\ \text{generated}}{\text{time}} = \frac{\text{QY}\times\text{photons absorbed}}{\text{time}} \qquad (3)$$

Here, we used the same excitation power for all wavelengths. The kinetic ratio at two different wavelengths is then

$$\frac{\text{rate}_{253.7\text{nm}}}{\text{rate}_{313\text{nm}}} = \frac{\text{QY}_{253.7\text{nm}}}{\text{QY}_{313\text{nm}}} \times \frac{\text{Abs}_{253.7\text{nm}}}{\text{Abs}_{313\text{nm}}} \qquad (4)$$

The quantum yields of ^{1}D oxygen generation are reported to be 0.133 at 253.7 nm and 0.020 at 313 nm[10]. The ratio of photon absorption equals the ratio of NaClO absorbance. Therefore,

$$\frac{\text{rate}_{253.7\text{nm}}}{\text{rate}_{313\text{nm}}} = \frac{0.133}{0.02} \times \frac{0.36}{0.64} = \frac{0.0484}{0.0129} \qquad (5)$$

The results are summarized in Supplementary Table 1. The O(^{1}D) photogeneration rates are plotted in Fig. 2b as a comparison to the doping rate constant. Taking zero as the reference point, the

action spectrum of doping rate constant matches the ratio of O (^{1}D) photogeneration rates at the two known wavelengths.

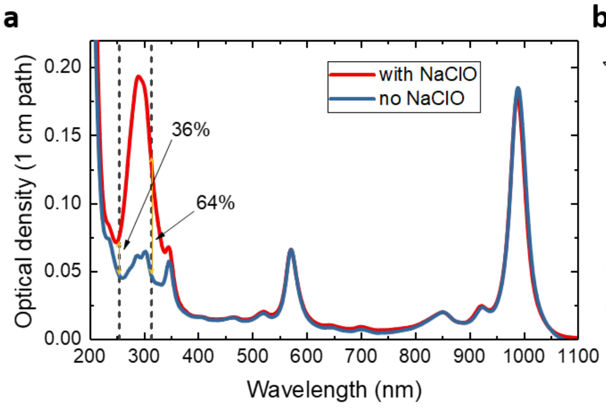

Supplementary Fig. 12 ^{1}D oxygen generation and doping rate constant. **a**, The absorption spectra of SWCNT solution with and without NaClO. The percentage of the photons absorbed by NaClO at 313 and 514.33 nm are listed. **b**, The doping rate constant with and without dissolved oxygen gas. The reaction is much faster when O_2 molecules have been removed. The reaction rate is also higher when the illumination wavelengths are shorter. Error bars are s.d. from spectral measurement noise.

Supplementary Table 1 The calculation of the ^{1}D oxygen photogeneration rates at two wavelengths

wavelength	quantum yield	absorbance	QY × Abs
313 nm	0.020 ± 0.015	0.64286 ± 0.00171	0.0129 ± 0.0096
253.7 nm	0.133 ± 0.017	0.36414 ± 0.00219	0.0484 ± 0.0060

Dissolved O_2 control. Here we purged the SWCNT solution with argon gas to remove dissolved oxygen molecules. Interestingly, the reaction rates increased significantly, proving that dissolved O_2 is not the reactant in the doping reaction. We suspect that the singlet oxygen atoms (^{1}D) were partially quenched by ground state oxygen molecules, slowing the doping reaction in the unpurged samples. Figure 2b uses the sample without O_2 to obtain accurate reaction kinetics, although in ambient conditions the reactions are efficient enough to run without purging. In summary, oxygen molecules in our doping reaction seem to have two side effects: first, they quench the singlet oxygen atoms that are essential for oxygen doping. Second, they slowly create fluorescent defects upon short wavelengths UV radiation, but with 10-120 times lower efficiency. The resulting product might also be different (E_{11}^* is ~1120 nm).

Energy diagram. The energies of several species were calculated using the PM3 semiempirical method and listed in Supplementary Table 2. The energy of a (6,5)-SWCNT segment nine hexagons in length was calculated to be −37392 kcal mol^{-1}. The ends were capped with H atoms in this simulation. A ±3 kcal mol^{-1} variation appears as the length varies from 7 to 18 hexagons. The binding energy of the ClO^- ions relative to an O atom and Cl^- ion is around 84.5 kcal $mole^{-1}$, which corresponds to a photon wavelength of 337 nm. The calculated binding energy is consistent with our illumination wavelengths. The original reactants, SWNT_6-5_L09 plus O-Cl^-, have a calculated energy of −37513.85 kcal mol^{-1}. The products, SWNT_6-5_L09_O_per plus Cl^-, have a total energy of −37541.22 kcal mol^{-1}, which is approximately 28 kcal mol^{-1} lower than the reactants. The epoxide adduct has energy similar to the reactants (−2.86 kcal mol^{-1}), thus that reaction channel is not energetically preferred. As expected, the Cl^- ion can be further stabilized in H_2O (H_2O energy is −217.22 kcal mol^{-1}). The solvation energy for Cl^- in a 7 H_2O system is −57

Supplementary Table 2 Examples of calculated energies of species calculated with PM3.

Structure	Energy (kcal)	Details	Comment
SWNT_6-5_L09	−37392.31	(6,5) SWCNT H-capped with length 9 hexagons	Calculated energy difference shown below was plus-minus 3 kcal for different SWCNT lengths from 7 to 18 hexagons
SWNT_6-5_L09_O_par	−37436.50	"Parallel" epoxide with O atom in the middle of the SWCNT	
SWNT_6-5_L09_O_per	−37461.01	"Perpendicular" ether, open ester structure, on the same SWCNT	About 25 kcal lower than epoxide
Cl−	−80.21	Cl (−) ion in vacuum	
O	43.16	Atom O in vacuum	Cl− + O is −37 kcal
O-Cl−	−121.54	O-Cl (−) ion in vacuum	84.5 kcal binding energy

Supplementary Table 3 The energy of ClO^- with different bond length in vacuum.

Length	1.702	1.8	1.9	2.0	2.1	2.2	2.3
Energy, kcal	−121.54	−118.73	−111.14	−100.80	−89.75	−79.48	−70.69
Length	2.4	2.5	2.6	2.7	2.8	2.9	3.0
Energy, kcal	−63.49	−57.71	−53.12	−49.48	−46.64	−44.45	−42.78
Length	3.1	3.2	3.3	3.4	3.5	3.6	3.7
Energy, kcal	−41.54	−40.63	−39.98	−39.52	−39.19	−38.96	−38.78
Length	3.8	3.9	4.0	4.5	5.0	6.0	7.0
Energy, kcal	−38.65	−38.54	−38.45	−38.09	−37.83	−37.51	−37.34

kcal mol^{-1}. In conclusion, we find that the most stable structure is formed when an oxygen atom dissociates from the ClO^- and bonds to the SWCNT to form the perpendicular ether adduct. The probability for this reaction occurring thermally is low because of the reaction barrier to O-Cl^- dissociation. Photoexcitation of the ClO^- ion overcomes this barrier. Also, stabilization of Cl^- by H_2O may stabilize the intermediate and accelerate the reaction.

Comparison to ozone method. The yields of ether-SWCNTs and epoxide-SWCNTs are related to their relative energies between reactants and products. Here, we use the stabilization energy, which is defined as the difference of total energies between products and reactants, to describe the thermodynamic preference. For example, the reactants of the oxygen doping in this work are SWCNT and ClO^- and the products of the reaction are either ether-SWCNT plus Cl^- or epoxide-SWCNT plus Cl^-. The stabilization energies then should be

$$\begin{cases} E_{\text{stab}}^{\text{ether}}(\text{ClO}^-) = E_{\text{SWCNT+ClO}^-} - E_{\text{ether}-\text{SWCNT+Cl}^-} \\ E_{\text{stab}}^{\text{epoxide}}(\text{ClO}^-) = E_{\text{SWCNT+ClO}^-} - E_{\text{epoxide}-\text{SWCNT+Cl}^-} \end{cases} \quad (6)$$

The $E_{\text{stab}}^{\text{ether}}(\text{ClO}^-)$ and $E_{\text{stab}}^{\text{epoxide}}(\text{ClO}^-)$ are 27.37 and 2.86 kcal mol^{-1}, respectively (shown in Supplementary Table 4). The total energy of the epoxide product is estimated to be only ca. 3 kcal mol^{-1} below that of the reactants (see table below). To further examine the product selectivity, we checked for the epoxide emission features in the spectra of Ghosh *et al.*[11] The extra sidebands in the range of 1,010 to 1,060 nm appeared in the first 5 hours, which might be from the E_{11}^{-} or E_{11}^{*+} emissions. But these less-stable forms seem to disappear after 16 hours. The authors attributed this to irreversible photoisomerization into more stable ether form. Therefore, the bulk of the O-SWCNT product apparently ended up in the ether form after some period of irradiation. By comparison, we did not observe significant emission sidebands other than E_{11}^{*} using the hypochlorite method, and our samples were not irradiated for a long time to allow photoisomerization. Therefore, we conclude that hypochlorite method has higher initial selectivity.

Supplementary Table 4 Comparison of stabilization energies using ozone and hypochlorite.

species	stabilization energy (kcal mol^{-1})	
	ether	epoxide
ozone	55	31
hypochlorite	27.37	2.86

Photodissociation of hypochlorite. Buxton *et al.*[10, 12] reported the photolysis of ClO^- ions into oxygen atom (^{3}P or ^{1}D) and chloride ion (Cl^-) under UV illumination at wavelengths of 253.7 nm,

313 nm, and 365 nm. Illumination at 365 nm produces only ground state oxygen atoms (^{3}P). We observed a low yield of O-doping with illumination at 360 nm, even though our simulation suggests that doping ground state oxygen atom onto SWCNT is also energy preferred. The more efficient reaction below 320 nm suggests that ^{1}D (excited) oxygen atoms play an important role in the doping process. Lim *et al.*[13] also showed that the negative charge of ClO^- ion redistributed from O to Cl when excited. However, the dissociation might redistribute the negative charge back to the oxygen atom when the structure is optimized. We cannot exclude the possibility of direct oxygen atom transfer from the excited ClO^- ion to SWCNT without full dissociation of ClO^-, although this mechanism seems inconsistent with the observation that dissolved O_2 suppresses the reaction rate.

Participation of exciton. One possible doping mechanism to consider is the involvement of hot excitons that have energy higher than E_{33}. However, hot nanotube excitons relax to their E_{11} state in ~100 fs[14], which suggests a very low probability for a hot exciton to encounter an O-doping agent. This would lead to a very inefficient reaction and long reaction times. If the reaction could be activated by ground state excitons, which have relaxation time up to ~100 ps, irradiation at 988 and 845 nm would give similar results as irradiation at 300 nm. This is not observed. Therefore, the results in Fig. 2a suggest the photo-dissociation of hypochlorite ions is essential to the doping mechanism.

O(^{1}D) quenching and doping yield. An isolated singlet oxygen atom O(^{1}D) has a very long radiative lifetime of ~114 s.[15] However, in practice its lifetime is far shorter and depends on chemical reactions with its environment. To our knowledge, measurements of the O(^{1}D) lifetime in aqueous solution have not been reported. Benedikt *et al.*[16] used plasma-generation to prove that oxygen atoms are highly stable in aqueous solution, showing no reaction with water, and are only quenched by encounters with reactive species. For example, the authors show an oxygen atom lifetime of 53 ns in 0.5 mM phenol aqueous solution. The 53 ns lifetime represents the mean diffusion time for oxygen atoms to meet a phenol molecule. The lifetime of oxygen in aqueous solution increased greatly to 32 μs when only dissolved O_2 was present as a quencher. This is consistent with a simulation result, which states that the O(^{3}P) remains stable in aqueous solution throughout the simulated time scale of 10 ps[17]. The authors also show that O(^{1}D) forms oxywater (H_2O--O) within the first iteration and remains stable throughout the rest of the simulation[17]. The conversion of oxywater into H_2O_2 was not observed in the simulation, probably due to the energy

barrier[18]. Therefore, it is reasonable to suppose that the O(^{1}D) atoms are stable in water until they reach a reactive species such as SWCNT or O_2. To further consider our reaction yield, we know that our optimal NaClO concentration is ~3 times higher than the concentration of nanotube carbon atoms. We can estimate that the average axial spacing between doping sites on an O-SWCNT product nanotube is ~100 nm, which corresponds to 8,800 carbon atoms. This would imply a NaClO-to-doping site ratio of 26,000. In other words, 26,000 hypochlorite ions would be needed to create one ether dopant site. This low efficiency suggests that most of the O(^{1}D) atoms are quenched by other reactive species, probably O_2 or surfactants. Therefore only the small fraction of O(^{1}D) atoms that are formed near nanotube sidewalls can successfully react with SWCNTs.

NaClO concentration. The basic SC surfactant gives the solution a pH of ~9.3, which is much higher than the 7.5 pK_a of $HClO/ClO^-$. Therefore, most of the hypochlorite exists in the form of ClO^- instead of HClO[19]. The Raman D/G ratio reveals the defect density of the treated SWCNTs. Judging by this ratio, NaClO at higher concentration creates more defects on the SWCNT walls. Figure 3c suggests that most of these defects are not fluorescent. NaClO at lower concentration creates fewer defects and a larger portion seem to be fluorescent quantum defects. The optimal concentration is around 0.1 mM but should vary slightly for each sample.

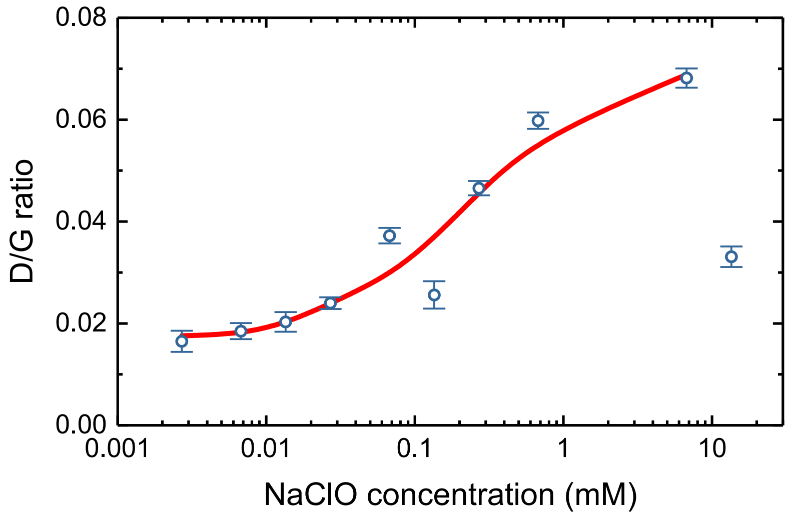

Supplementary Fig. 13 D/G Raman ratio vs NaClO concentration. The samples are the same as those in Fig. 3c. Error bars are s.d. from spectral measurement noise.

Variance spectroscopy. Variance spectroscopy measures fluctuations of SWCNT emission spectra, from which many results can be obtained. One of these is the relative abundance spectrum, expressed as the ratio of mean spectrum divided by the variance spectrum:

$$N(\lambda) = \frac{\langle I(\lambda)\rangle^2}{\sigma^2(\lambda)} \tag{7}$$

The mean emission intensity per particle spectrum (relative emission efficiencies) then can be written as

$$\varepsilon(\lambda) = \frac{\langle I(\lambda)\rangle}{N(\lambda)} = \frac{\sigma^2(\lambda)}{\langle I(\lambda)\rangle} = \frac{\sigma(\lambda)}{\sqrt{N(\lambda)}} \tag{8}$$

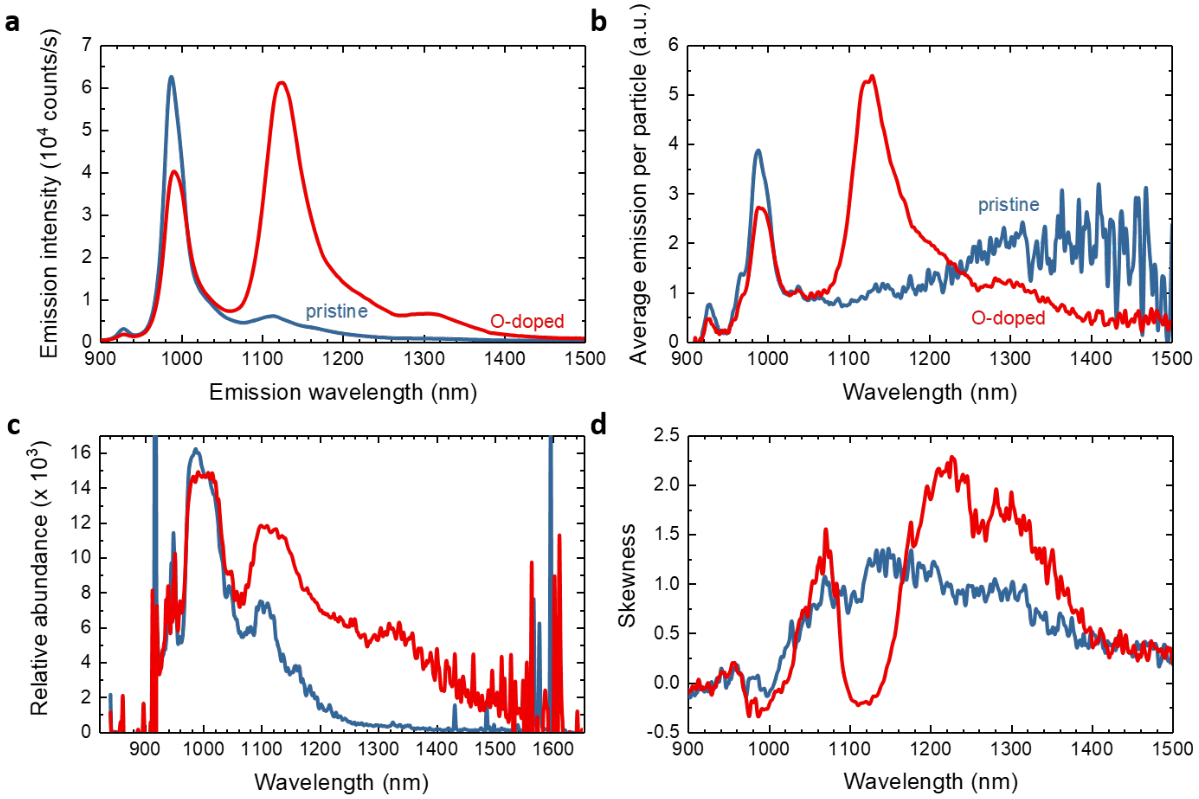

Supplementary Fig. 14 Various types of spectra measured from Variance Spectrometer. **a**, Mean spectra. **b**, Emission efficiency spectra. **c**, Relative abundance spectra. **d**, Skewness spectra.

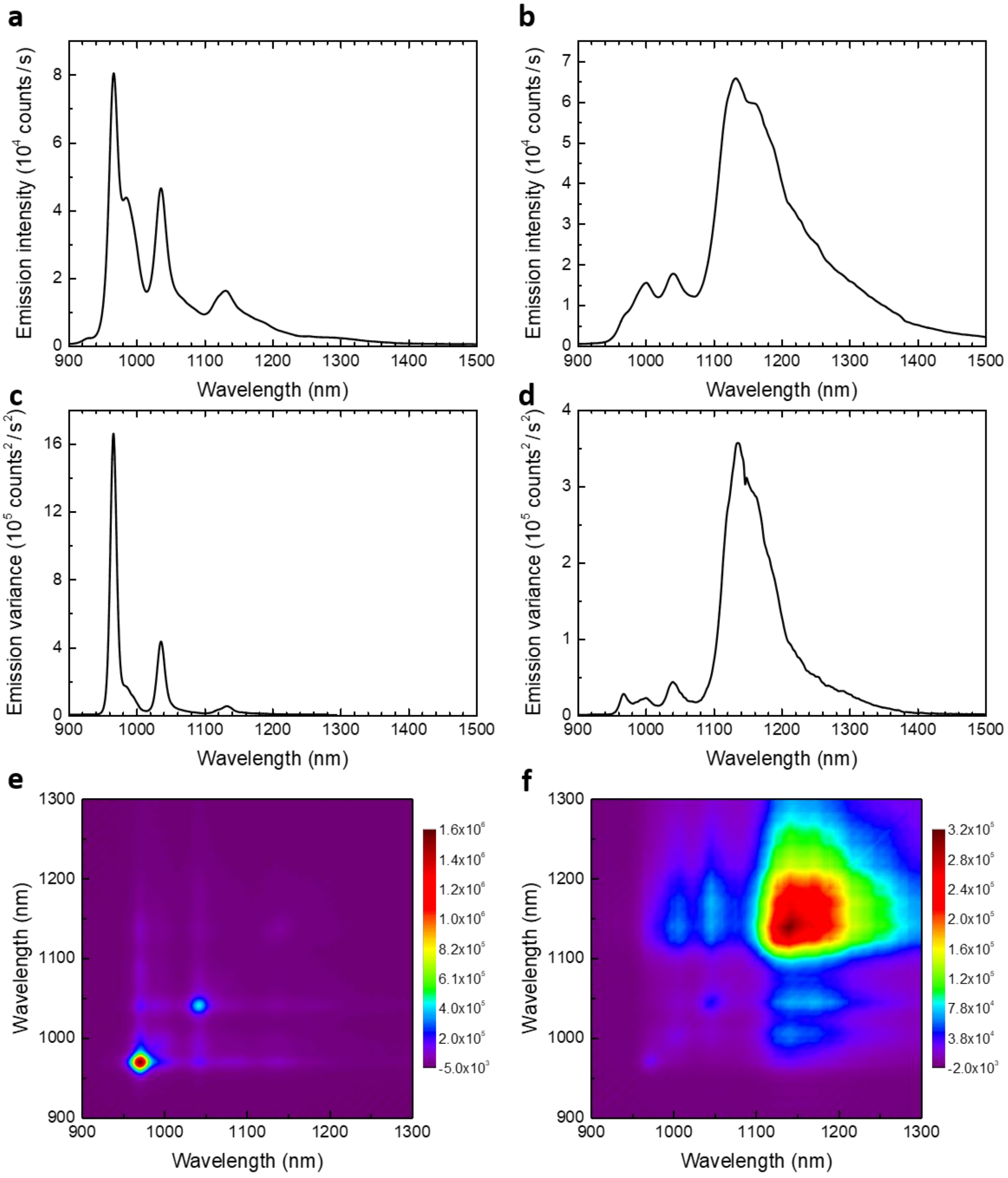

Supplementary Fig. 15 Variance spectroscopy of pristine and O-doped CoMoCAT SWCNTs excited at 660 nm. **a**, Mean spectrum of pristine SWCNTs, **b**, Mean spectrum of O-doped SWCNTs. **c**, Variance spectrum of pristine SWCNTs. **d**, Variance spectrum of O-doped SWCNTs. **e**, Covariance matrix of pristine SWCNTs. **f**, Covariance matrix of O-doped SWCNTs.

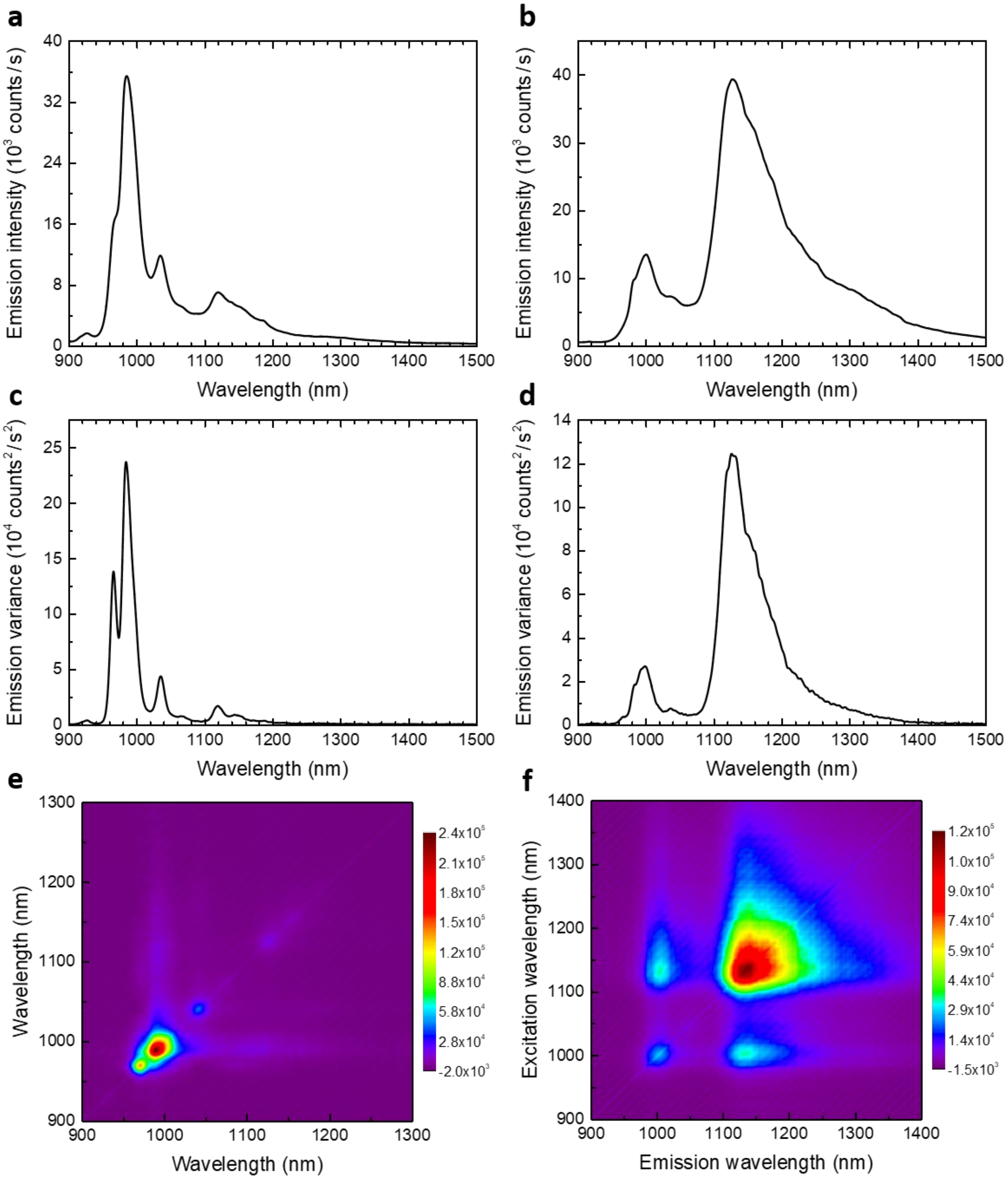

Supplementary Fig. 16 Variance spectroscopy of pristine and O-doped CoMoCAT SWCNTs excited at 785 nm. **a**, Mean spectrum of pristine SWCNTs, **b**, Mean spectrum of O-doped SWCNTs. **c**, Variance spectrum of pristine SWCNTs. **d**, Variance spectrum of O-doped SWCNTs. **e**, Covariance matrix of pristine SWCNTs. **f**, Covariance matrix of O-doped SWCNTs.

Calculation of Pearson correlation coefficient and doping distribution. Assume that two emissive components, E_{11} and E_{11}^*, are seen from treated (6,5)-SWCNT samples. It is possible that some of the nanotubes remain undoped and show pristine E_{11} emission profiles, while others are so heavily doped so that no E_{11} emission can be observed. The other possibility is that individual SWCNTs can emit in both bands. We can deduce the fractions of these three situations using the following derivations. The Pearson correlation coefficient can be expressed as the following function[20]

$$\rho_{\lambda_j}(\lambda_k) = \sqrt{\frac{n_k^0}{n_j^0}}\frac{\mathrm{cov}(\lambda_j,\lambda_k)}{\sigma(\lambda_j)\sigma(\lambda_k)} \tag{9}$$

where $\sigma(\lambda)$ is the variance at wavelength λ, $\mathrm{cov}(\lambda_j, \lambda_k)$ is the covariance of λ_k and λ_j, and $\left(n_k^0/n_j^0\right)^{1/2}$ accounts for differing initial abundances of the two components.

$$\rho_{\lambda_j}(\lambda_k) = \sqrt{\frac{n_k^0}{n_j^0}}\frac{\mathrm{cov}(\lambda_j,\lambda_k)}{\sigma^2(\lambda_j)}\frac{\sigma(\lambda_j)}{\sigma(\lambda_k)} \tag{10}$$

$$\rho_{\lambda_j}(\lambda_k) = \frac{\mathrm{cov}(\lambda_j,\lambda_k)}{\sigma^2(\lambda_j)}\frac{\sigma(\lambda_j)/\sqrt{n_j^0}}{\sigma(\lambda_k)/\sqrt{n_k^0}} = \frac{\mathrm{cov}(\lambda_j,\lambda_k)}{\sigma^2(\lambda_j)}\frac{\varepsilon(\lambda_j)}{\varepsilon(\lambda_k)} = \frac{\mathrm{cov}(\lambda_j,\lambda_k)/\sigma^2(\lambda_j)}{\varepsilon(\lambda_k)/\varepsilon(\lambda_j)} \tag{11}$$

Therefore, the Pearson correlation coefficient or Pearson's *r* can be written as

$$\boxed{\rho_{\lambda_j}(\lambda_k) = \frac{\text{covariance of } \lambda_j \text{ and } \lambda_k \text{ normalized to variance of } \lambda_j}{\lambda_k \text{ to } \lambda_j \text{ emission efficiency ratio}} = \frac{\mathrm{cov}_{\lambda_j}(\lambda_k)}{e_{\lambda_j}(\lambda_k)}} \tag{12}$$

The Pearson correlation coefficient spectra at E_{11} and E_{11}^* ($\rho_{994\mathrm{nm}}(\lambda)$ and $\rho_{1126\mathrm{nm}}(\lambda)$) are plotted in the main text Fig. 4d. More complete displays of the Pearson correlation coefficients relative to each wavelength are shown in Supplementary Fig. 17e,f as Pearson matrices. In Supplementary Fig. 17e, for the pristine SWCNT sample, only one major band is shown around 994 nm. A minor band near 1100 nm represents the E_{11} sideband emission. For comparison, the plot in Supplementary Fig. 17f refers to the O-doped SWCNT sample. Two major bands at 994 nm and 1126 nm reveal the strong correlation between the E_{11} and E_{11}^* emissions. A minor band at ~1320 nm might be assigned as a sideband of the E_{11}^* transition, which is also discussed in the previous section (See Supplementary Fig. 2b). To estimate the correlation between the E_{11} and E_{11}^* emissions, we simply use the peak positions at 994 nm for E_{11} and 1260 nm for E_{11}^*. Therefore, the Pearson correlation coefficients are

$$\begin{cases} \rho_{994\mathrm{nm}}(1126\mathrm{nm}) = 0.7251 \\ \rho_{1126\mathrm{nm}}(994\mathrm{nm}) = 0.9066 \end{cases} \tag{13}$$

They can be interpreted as: about 91% of the E_{11}^{*} emitting SWCNTs also have E_{11} emission and about 73% of the E_{11} emitting SWCNTs also have E_{11}^{*} emission. Assume that there are three types of SWCNTs after doping: E_{11} only, E_{11}^{*} only and $E_{11} + E_{11}^{*}$. We want to know the fraction of each type of SWCNTs, which are $f_{E_{11}^{\text{only}}}$, $f_{E_{11}^{*,\text{only}}}$ and $f_{E_{11}+E_{11}^{*}}$, respectively. Their numbers are $N_{E_{11}^{\text{only}}}$, $N_{E_{11}^{*,\text{only}}}$ and $N_{E_{11}+E_{11}^{*}}$. The total number of SWCNTs that have E_{11} emission is $N_{E_{11}}$, and the total number of SWCNTs that have E_{11}^{*} emission is $N_{E_{11}^{*}}$. We then have the following relationship:

$$\begin{cases} N_{E_{11}} = N_{E_{11}+E_{11}^{*}} + N_{E_{11}^{\text{only}}} \\ N_{E_{11}^{*}} = N_{E_{11}+E_{11}^{*}} + N_{E_{11}^{*,\text{only}}} \end{cases} \tag{14}$$

The definition of the Pearson correlation coefficient in our case is

$$\begin{cases} \rho_{E_{11}^{*}}(E_{11}) = \frac{N_{E_{11}^{*}}}{N_{E_{11}+E_{11}^{*}}} \\ \rho_{E_{11}}(E_{11}^{*}) = \frac{N_{E_{11}}}{N_{E_{11}+E_{11}^{*}}} \end{cases} \tag{15}$$

Therefore, the number of SWCNTs that contain both E_{11} and E_{11}^{*} emissions can be calculated

$$N_{E_{11}+E_{11}^{*}} = \rho_{E_{11}^{*}}(E_{11}) N_{E_{11}^{*}} = \rho_{E_{11}}(E_{11}^{*}) N_{E_{11}} \tag{16}$$

Because there are only three types of SWCNTs, the total number of SWCNTs is

$$N_{\text{total}} = N_{E_{11}^{\text{only}}} + N_{E_{11}^{*,\text{only}}} + N_{E_{11}+E_{11}^{*}} \tag{17}$$

This can be reformulated into fraction

$$f_{E_{11}^{\text{only}}} + f_{E_{11}^{*,\text{only}}} + f_{E_{11}+E_{11}^{*}} = \frac{N_{E_{11}^{\text{only}}}}{N_{\text{total}}} + \frac{N_{E_{11}^{*,\text{only}}}}{N_{\text{total}}} + \frac{N_{E_{11}+E_{11}^{*}}}{N_{\text{total}}} = 1 \tag{18}$$

The fraction of each type of SWCNTs can be calculated

$$\begin{cases} f_{E_{11}^{\text{only}}} = \frac{N_{E_{11}^{\text{only}}}}{N_{\text{total}}} = \frac{1}{1+\frac{1}{\frac{N_{E_{11}}}{N_{E_{11}^{*}}}\left[1-\rho_{E_{11}}(E_{11}^{*})\right]}} \\ f_{E_{11}^{*,\text{only}}} = \frac{N_{E_{11}^{*,\text{only}}}}{N_{\text{total}}} = \frac{1}{1+\frac{N_{E_{11}}/N_{E_{11}^{*}}}{1-\rho_{E_{11}^{*}}(E_{11})}} \\ f_{E_{11}+E_{11}^{*}} = \frac{N_{E_{11}+E_{11}^{*}}}{N_{\text{total}}} = \frac{\rho_{E_{11}^{*}}(E_{11})}{\left[1-\rho_{E_{11}^{*}}(E_{11})\right]+\frac{N_{E_{11}}}{N_{E_{11}^{*}}}} \end{cases} \tag{19}$$

The Pearson correlation coefficients of E_{11} and E_{11}^{*} ($\rho_{E_{11}}(E_{11}^{*})$ and $\rho_{E_{11}^{*}}(E_{11})$) can be estimated from their peak emissions ($\rho_{994\text{nm}}(1126\text{nm})$ and $\rho_{1126\text{nm}}(994\text{nm})$), which are 0.7251 and 0.9066 respectively. The ratio $N_{E_{11}}/N_{E_{11}^{*}}$, which can be obtained from relative abundance spectrum, is 14718/11591=1.2698. Therefore, the fractions are

$$
\begin{cases}
F_{\mathrm{E}_{11}^{\mathrm{only}}} = \dfrac{N_{\mathrm{E}_{11}^{\mathrm{only}}}}{N_{\mathrm{total}}} = \dfrac{1}{1+\frac{1}{1.2698\times[1-0.7251]}} = 0.259 \\
F_{\mathrm{E}_{11}^{*,\mathrm{only}}} = \dfrac{N_{\mathrm{E}_{11}^{*,\mathrm{only}}}}{N_{\mathrm{total}}} = \dfrac{1}{1+\frac{1.2698}{[1-0.9066]}} 0.069 \\
F_{\mathrm{E}_{11}+\mathrm{E}_{11}^{*}} = \dfrac{N_{\mathrm{E}_{11}+\mathrm{E}_{11}^{*}}}{N_{\mathrm{total}}} = \dfrac{0.9066}{1-0.9066+1.2698} = 0.665
\end{cases}
\tag{20}
$$

For this specific sample, we find that 26% of the SWCNTs are not doped with oxygen and 7% of the SWCNTs are so heavily doped that no E_{11} emission can be detected. The rest of them have both E_{11} and E_{11}^{*} emissions. Relative abundance and emission efficiencies used in the calculation can be obtained from Supplementary Fig. 14.

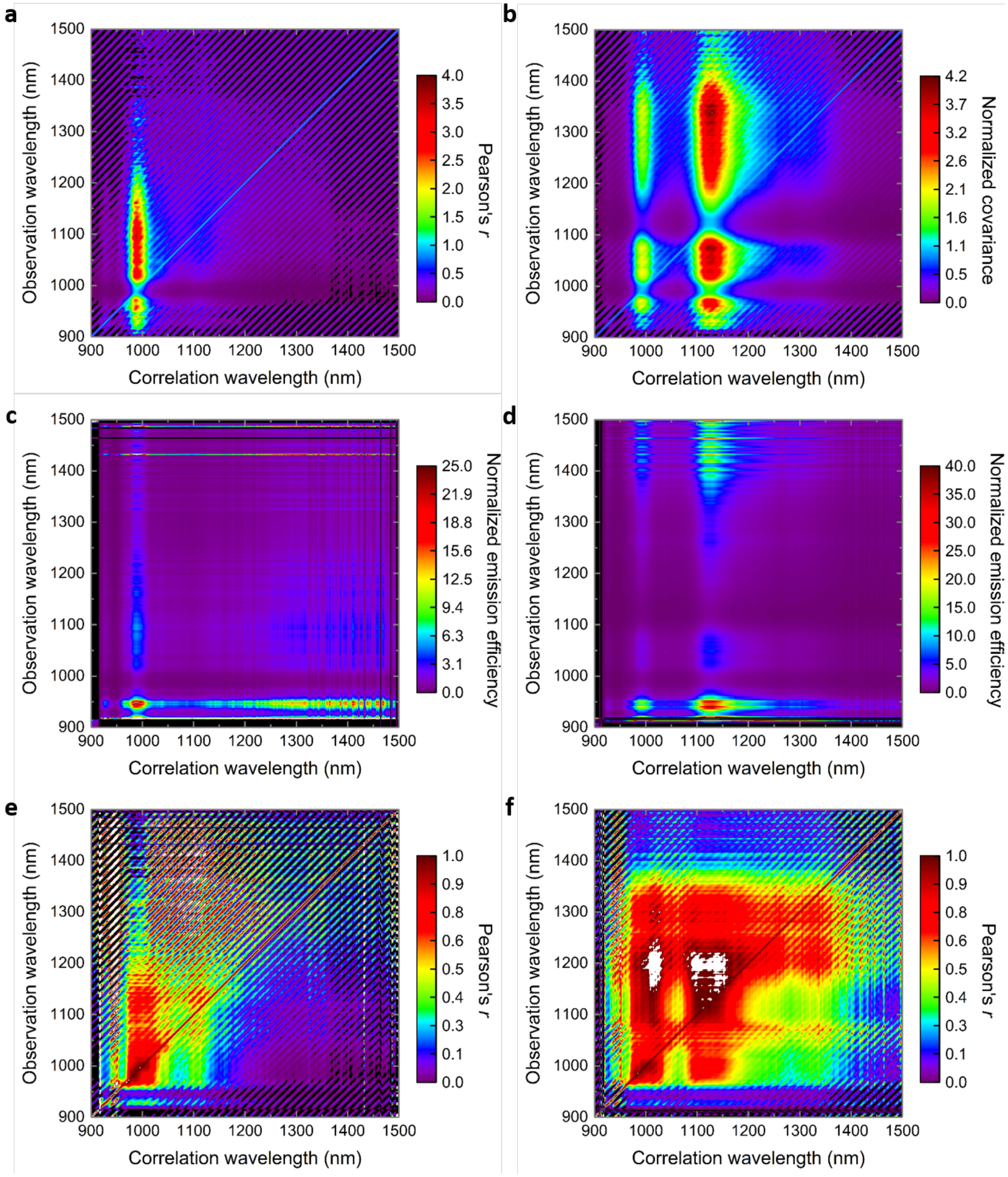

Supplementary Fig. 17 Normalized covariance, normalized emission efficiency and Pearson correlation coefficient matrix. **a**, Pearson correlation coefficient matrix of pristine (6,5)-SWCNTs. **b**, Pearson correlation coefficient matrix of O-doped (6,5)-SWCNTs. **c**, Normalized emission efficiency matrix of pristine (6,5)-SWCNTs. **d**, Normalized emission efficiency matrix of O-doped (6,5)-SWCNTs. **e**, Pearson correlation coefficient matrix of pristine (6,5)-SWCNTs. **f**, Pearson correlation coefficient matrix of O-doped (6,5)-SWCNTs.

E_{11}^{*} assignment of right and left handed (6,5)-SWCNTs. The existence of different E_{11}^{*} emission wavelengths for –(6,5) and +(6,5) enantiomers is clear evidence that the E_{11}^{*} emission is affected by the environment. It has been reported[11] that the E_{11} emission of –(6,5) is blue-shifted relative to +(6,5) in a chiral cholate coating. Here, we have prepared a pure –(6,5) sample based on the published sorting method[1] and doped with oxygen to examine the wavelength shift. As shown in Supplementary Fig. 18, the E_{11}^{*} emission band from the –(6,5) enantiomers is also slightly blue-shifted, matching the shift direction of the E_{11} emission.

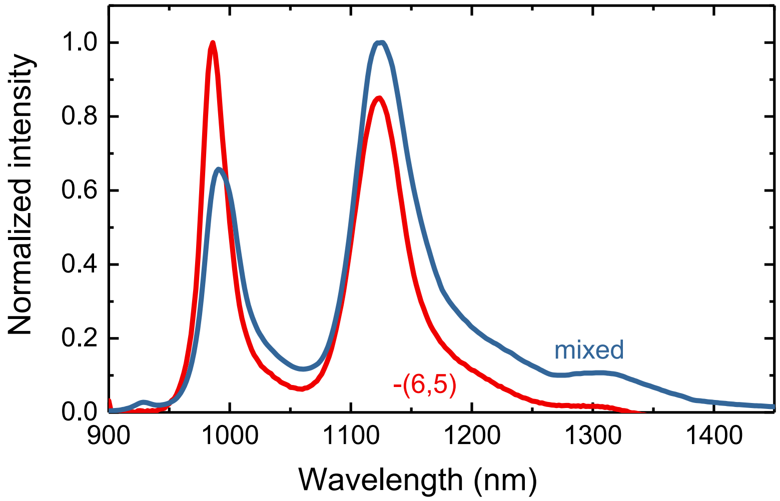

Supplementary Fig. 18 The E_{11} and E_{11}^{*} peak shifts of the –(6,5) SWCNTs.

Another doped sample for variance spectroscopy.

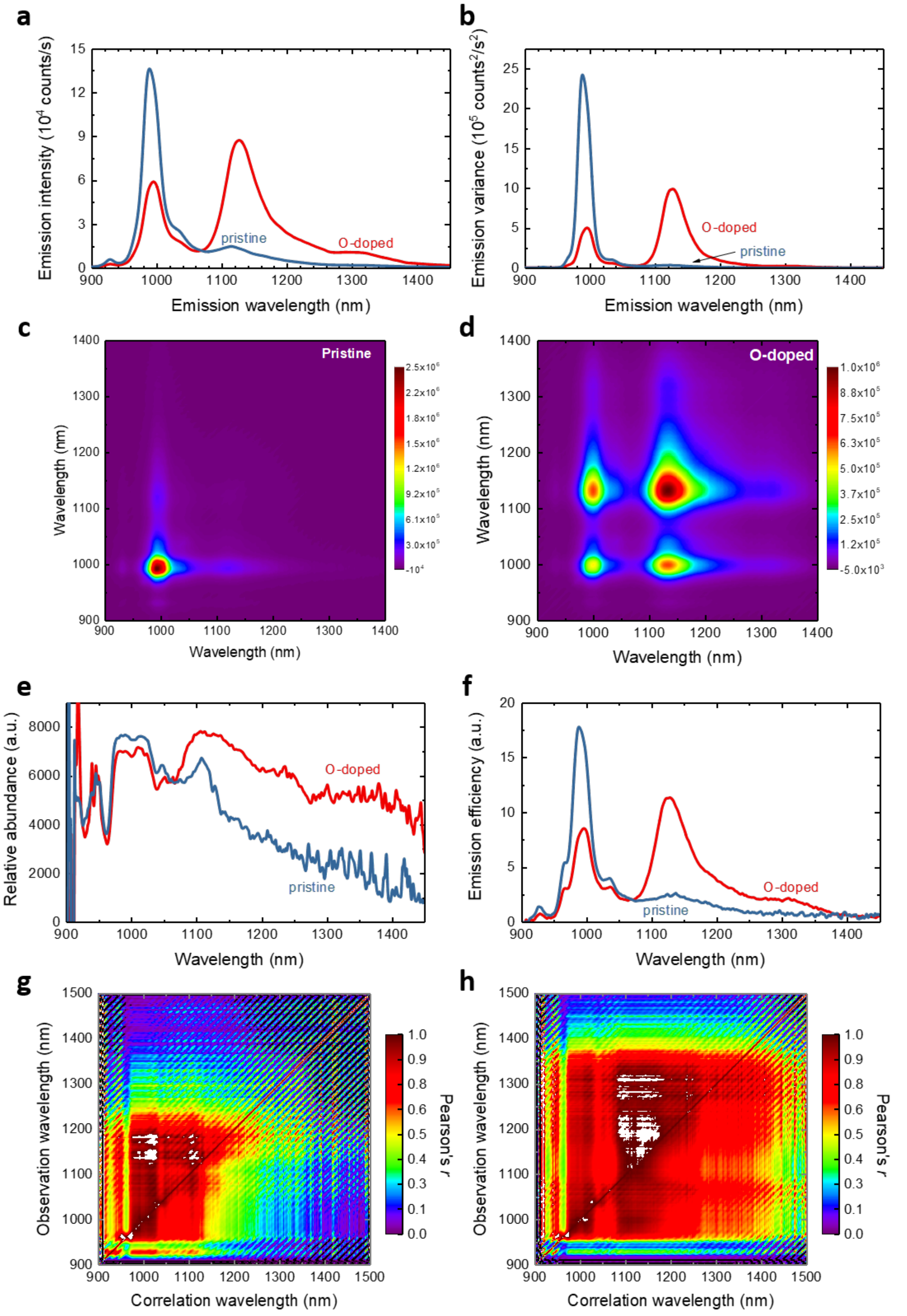

Supplementary Fig. 19 Variance spectroscopy of sample 2. **a**, Mean spectra. **b**, Variance spectra. **c**, Covariance matrix of pristine (6,5)-SWCNTs. **d**, Covariance matrix of O-doped (6,5)-SWCNTs. **e**, Relative abundance spectra. **f**, Emission efficiency **g**, Pearson correlation coefficient matrix of pristine (6,5)-SWCNTs. **h**, Pearson correlation coefficient matrix of O-doped (6,5)-SWCNTs.

Doping extent vs doping homogeneity. The doping extent may not indicate the doping homogeneity of the sample. Here, we compare two samples with very similar doping extents and estimate their doping homogeneity using relative abundance data and Pearson correlation coefficients. As shown in Supplementary Fig. 20a, the mean spectra of the two treated samples are nearly identical. However, the variance E_{11}^{*} peak (see Supplementary Fig. 20b) is much higher for sample 1 than for sample 2. The percentage of SWCNTs that remain undoped is lower for sample 2, indicating a more homogeneous distribution of the O-doping sites (Supplementary Fig. 20c). For applications in fluorescence imaging, a minimum value of E_{11}^{only} is desired to obtain the maximum E_{11}^{*} emission per SWCNT dose. However, for applications in single photon emission, one might want minimum $E_{11}^{*,only}$ emission because only one doping site is required for each SWCNT. Variance spectroscopy helps to characterize sample suitability for such applications.

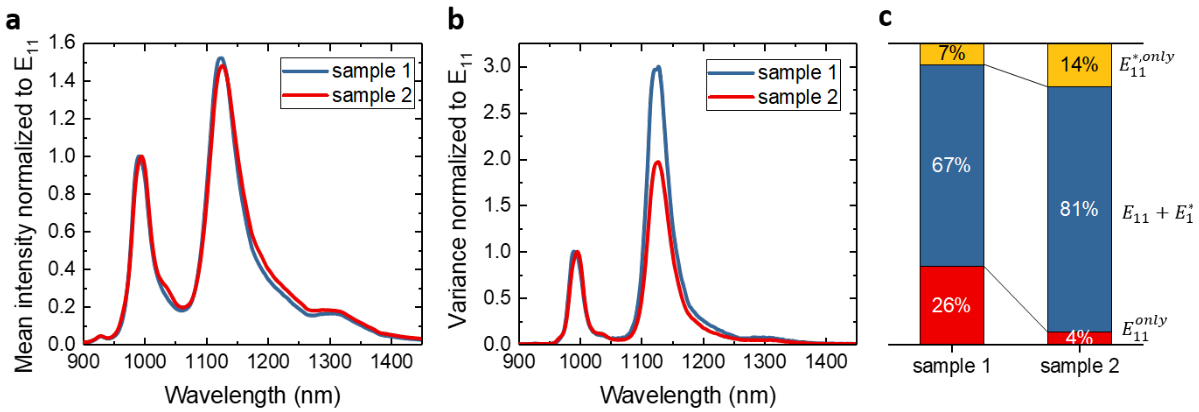

Supplementary Fig. 20 Doping extent vs doping heterogeneity. **a**, Mean spectra normalized to E_{11}. **b**, Variance spectra normalized to E_{11}. **c**, The percentage of each type of SWCNTs.

Calibration of pixel size. We calibrated the pixel size for imaging experiments using a 1951 USAF Target. The density of the lines is 114 black-white pairs per mm. The measured line pair is 17.691 pixels, which corresponds to 496 nm per pixel.

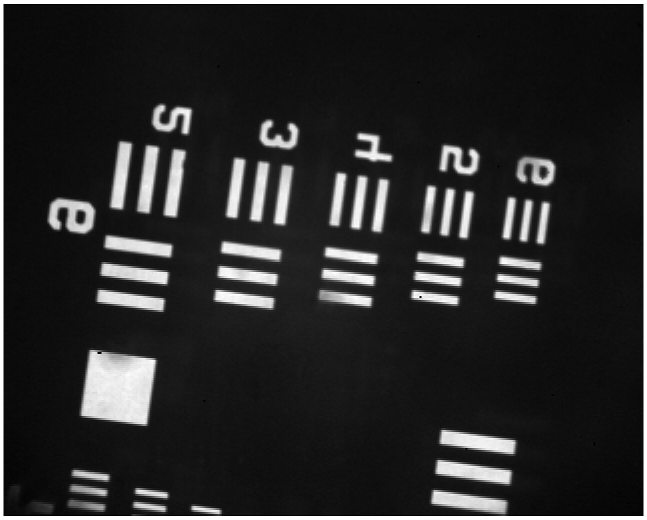

Supplementary Fig. 21 Pixel size measurement.

Single particle measurements. Both pristine and O-doped (6,5)-SWCNTs were dispersed on cover slips and fluorescence micrographs were taken using two sets of filters. Here, channel 1 represents the spectral window from 950 to 1000 nm (Thorlabs filters FELH950 + FESH1000) and channel 2 represents the spectral window from 1100 to 1300 nm (filters Edmunds OD4 1100LP + OD4 1300SP). The SWCNTs were excited at 850 nm with a MaiTai laser system (Spectra Physics). The laser was transmitted to the microscope system using a high power optical fiber, giving a depolarized output beam. A 40x NIR objective (Zeiss LD C-Apochromat) was used to focus the excitation and collect the emission. The emission was refocused into the InGaAs camera using a tube lens (Thorlabs TTL200-S8). The camera was operated at high gain and 5 MHz ADC conversion rate. Its frame time was set to 50 ms and a 1000-frame video was recorded to obtain an averaged image. Because the pixel size was ~500 nm (see Supplementary Fig. 21) and most of the SWCNTs had lengths shorter than the pixel size, we simply use the maximum intensity from each single pixel to represent the SWCNT emission intensity (Supplementary Fig. 22a). Supplementary Fig. 22b shows the intensity ratio vs intensity sum for all detected SWCNTs. The intensity sum is the total intensities from channels 1 and 2, and the intensity ratio is the ratio of channel 2 to channel 1 intensity. Some SWCNTs show bright emission in one channel but are invisible in the other. We use the noise level to estimate the upper limit for intensities of the invisible tubes, which are displayed as data points in light colors (light blue and light red). These points have underestimated or overestimated intensity ratios, depending on which channel is invisible. As discussed in the main text, the intensity ratio difference between O-doped and pristine SWCNTs is larger for SWCNTs having larger intensity sums. This suggests that the longer SWCNTs have better doping efficiency. We interpret this as an indication of relatively homogeneous doping on the SWCNT walls. Supplementary Fig. 22c shows the probability distribution of intensity ratios of O-doped and pristine SWCNTs. As expected, the pristine SWCNTs with and without the presence of NaClO are very similar, indicating an absence of significant dark reaction. The O-doped SWCNTs have notably higher intensity ratios, but some of the lower values overlap with the higher ratio values of pristine SWCNTs. These overlapped intensity ratio values are from shorter SWCNTs, as shown in Supplementary Fig. 22b.

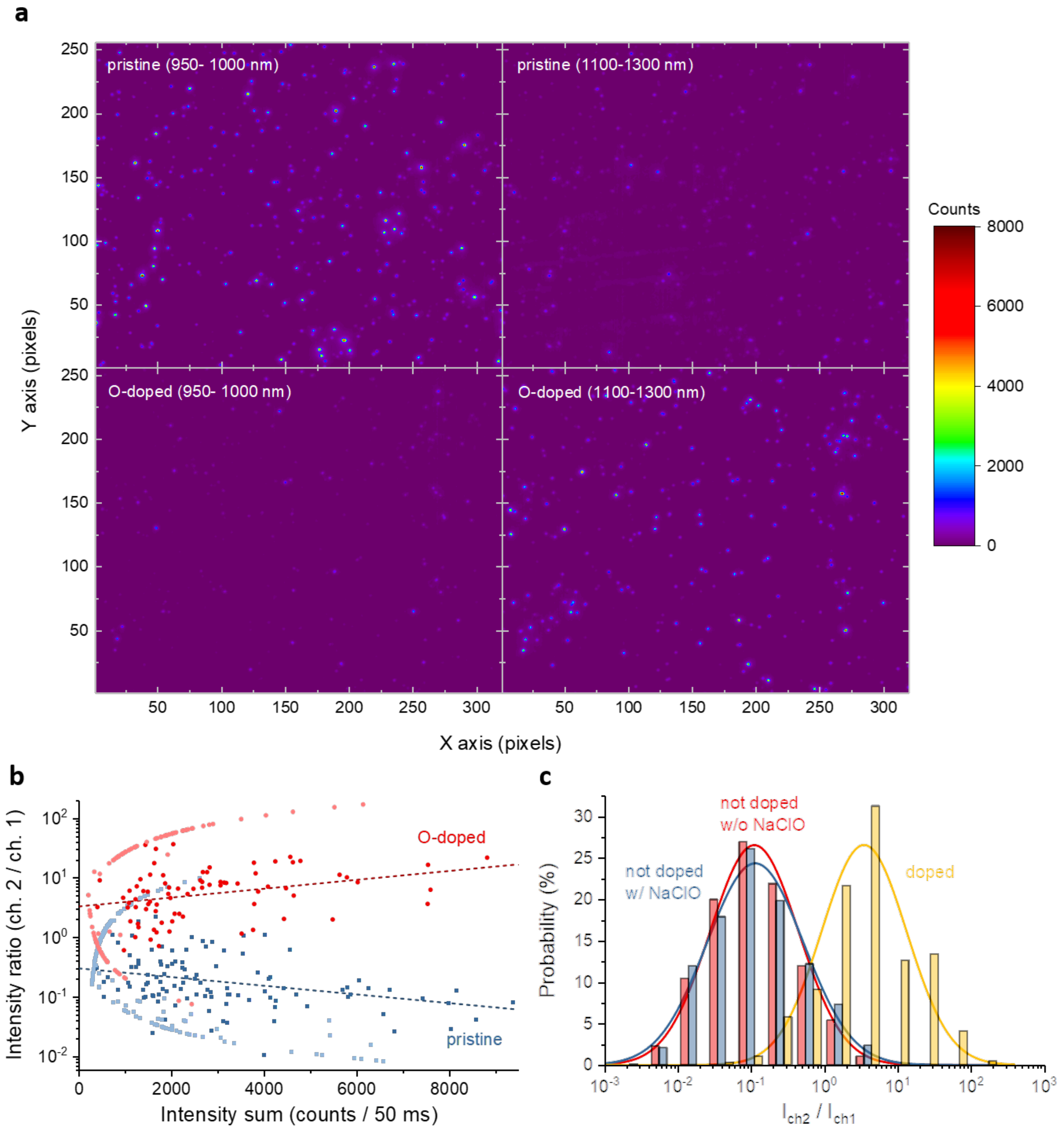

Supplementary Fig. 22 Single nanotube measurement. **a**, Raw SWIR images of nanotubes on coverslip surface. The images are acquired at two different wavelength channels (Ch. 1: 950 - 1000 nm; Ch. 2: 1100 - 1300 nm). The pixel size is ~500 nm. **b**, Intensity ratio vs intensity sum. Light red circles and light blue squares are the SWCNTs with intensity in one of the channels lower than detection limit. **c**, The distribution of the intensity ratios of pristine and O-doped SWCNTs deduced from Supplementary Fig. 22a.

Other water-soluble oxidizing agents. Because the O-doping is an oxidative process, we investigated whether other water soluble oxidizing agents could give similar results. Ghosh *et al.*[11] demonstrated that reaction with ozone could dope oxygen atoms into SWCNTs, but controlling for accurate and reproducible ozone concentration in liquid is challenging. Chiu *et al.*[21] utilized the auto-oxidation of linoleic acid to produce peroxide in solution. The authors showed efficient oxygen doping, but the amount of peroxide produced from auto-oxidation is also difficult to control. Therefore, the use of simple water soluble oxidizing agents, instead of gases or low solubility compounds, might give promising results. Supplementary Fig. 23 shows O-doping using several strong oxidizing agents listed in the order of their standard reduction potential at pH 9.3. The reactions are examined with several different illumination wavelengths (on and off the absorption peaks of the oxidizing agents) and we only show the data with the highest doping results. The order of doping extent matches the order of reduction potentials of the oxidizing agents except in the case of $S_2O_8^{2-}$ ions. We suspect that the doping reaction needs direct donation of oxygen atoms from the oxidizing agents. $K_2Cr_2O_7$ is a very strong oxidizing agent in acidic solution ($E^0 = 1.33$) but decomposes into CrO^{4-} in basic solution. Therefore, no oxygen doping was observed using $K_2Cr_2O_7$. Similarly, H_2O_2 is a very strong oxidizing agent in acidic solution but shows lower reduction potential in basic solution. The reaction rate is slower, and the yield is lower compared to ClO^-. Tuning the acidity of the solution for higher reduction potential is not practical here because it also greatly affects the surfactants coatings and leads to faster nanotube aggregation. Another factor that decreases the doping efficiency of H_2O_2 is low absorption. $KMnO_4$ shows acceptable doping density and reaction rate with 250 nm and 350 nm irradiation. It is worth mentioning that $KMnO_4$ quenches SWCNT fluorescence (before and after illumination). Adding extra SDC surfactant is necessary for fluorescence recovery. Using $KMnO_4$, the doping can proceed on a slower time scale (~10 min) with longer irradiations wavelengths up to 500 nm (Supplementary Fig. 23 & 25). More detailed studies of the reaction mechanism are necessary. The formation of MnO_2 nanoparticles (brownish observed color) during the reaction process makes the solution dirtier and harder to clean. We find that oxygen doping using NaClO gives the highest E_{11}^*/E_{11} ratio and the largest $\phi_{\text{O-doped}}/\phi_{\text{pristine}}$, demonstrating the best doping quality. We suspect that efficient doping requires "direct" donation of singlet oxygen atom very close to the SWCNT surface. In this view, the simple structure of hypochlorite ions is an advantage. The high reduction potential of ClO^- at high pH is ideal.

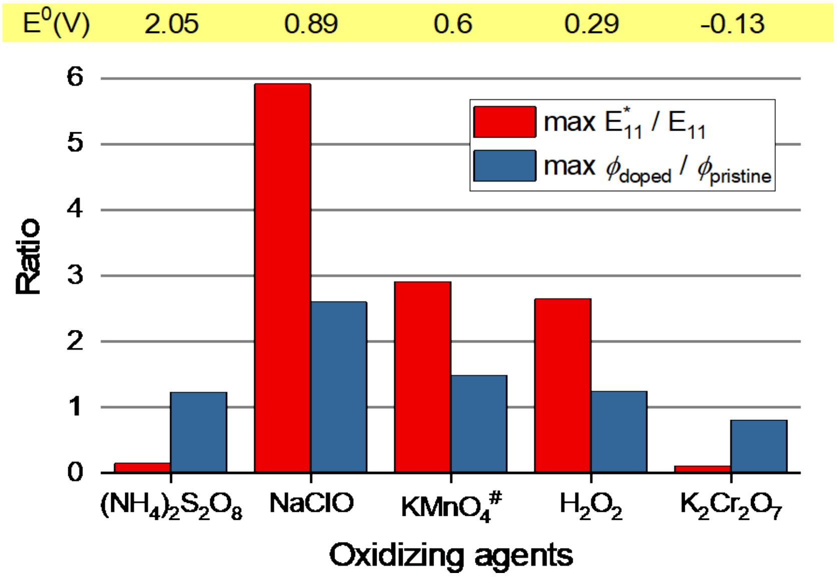

Supplementary Fig. 23 O-doping using other water-soluble oxidizing agents. ϕ represents the quantum yield.

SWCNT oxidation in dark. Pristine SWCNTs are stable structures that require harsh conditions to destroy. Researchers have used strong oxidizing agents at high temperature to modify the SWCNT side walls. Here, we examined the oxidative effects of several strong water-soluble oxidizing agents on the SWCNT structure. Approximately 1 mM of oxidizing agent was added to (6,5)-enriched SWCNT suspensions in 0.07% SC and the samples were then left in the dark for 24 h. Supplementary Fig. 24 shows the Raman spectra of the SWCNT samples after 24 h of incubation. The D/G ratio remained the same, indicating no modification of SWCNT side wall when isolated from light. Fluorescence spectra confirmed this result.

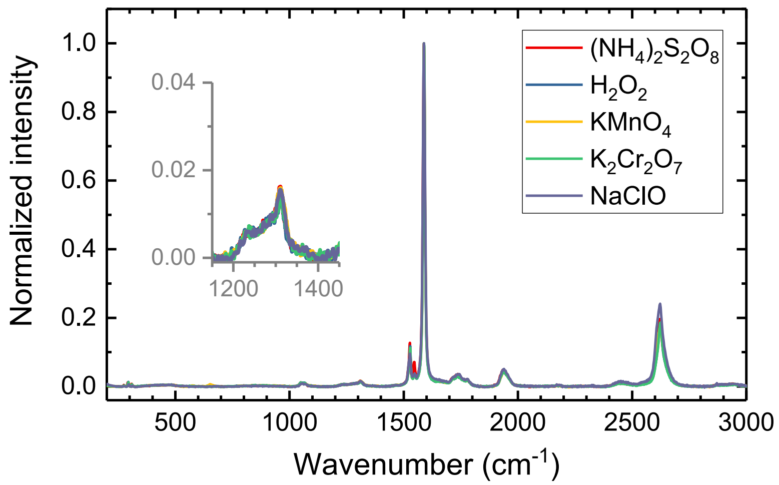

Supplementary Fig. 24 The Raman spectra of (6,5)-SWCNTs after left in the dark after 24 h.

Oxygen doping using $KMnO_4$. The permanganate ion is known to give an oxygen atom upon photo-excitation[22, 23]. One of the resulting products is the MnO_2 nanoparticles. We observed the sample color change from purple to yellow after irradiation. The MnO_4^- ions quench SWCNT fluorescence in SC suspensions. Therefore, similar to the reaction in SDS surfactants, we could not monitor the reaction during the doping steps and had to add SDC to restore the fluorescence. We found that the reaction rate was similar for near-UV irradiation but became slower for longer wavelengths. Here, we demonstrate good O-doping of SWCNTs using $KMnO_4$. The advantage of using $KMnO_4$ is that the reaction can proceed with irradiation by visible wavelengths, even though the reaction rate is slower. The disadvantage is the generation of MnO_2 nanoparticles. This might require more complex post-processing to remove those unwanted side products.

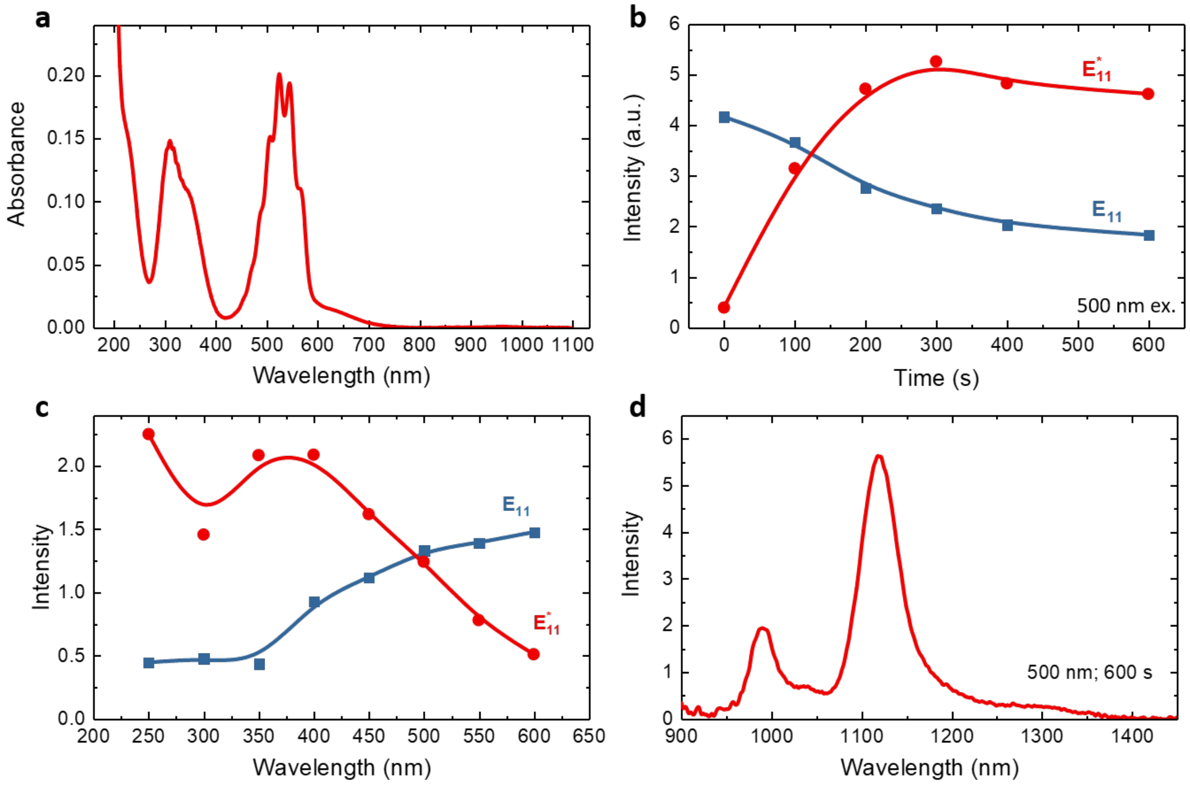

Supplementary Fig. 25 Oxygen doping of CoMoCAT SWCNTs using $KMnO_4$. **a**, Absorption spectrum of $KMnO_4$. **b**, Reaction kinetics excited at 500 nm. **c**, Action spectra of E_{11} and E_{11}^* peaks. **d**, Fluorescence spectrum after oxygen doping.

Oxygen doping using H_2O_2. The photo-decomposition of H_2O_2 can also produce oxygen atoms[24]. However, the products of irradiated H_2O_2 seem to destroy the SWCNT structure. We find that the resulting SWCNT fluorescence intensity is always lower than that of the SWCNTs doped by NaClO and $KMnO_4$. Also, the reaction rate is much slower compared to NaClO and $KMnO_4$ reactions, probably because the extinction coefficient of H_2O_2 is much lower (~18.4 $M^{-1}cm^{-1}$ at 254 nm).

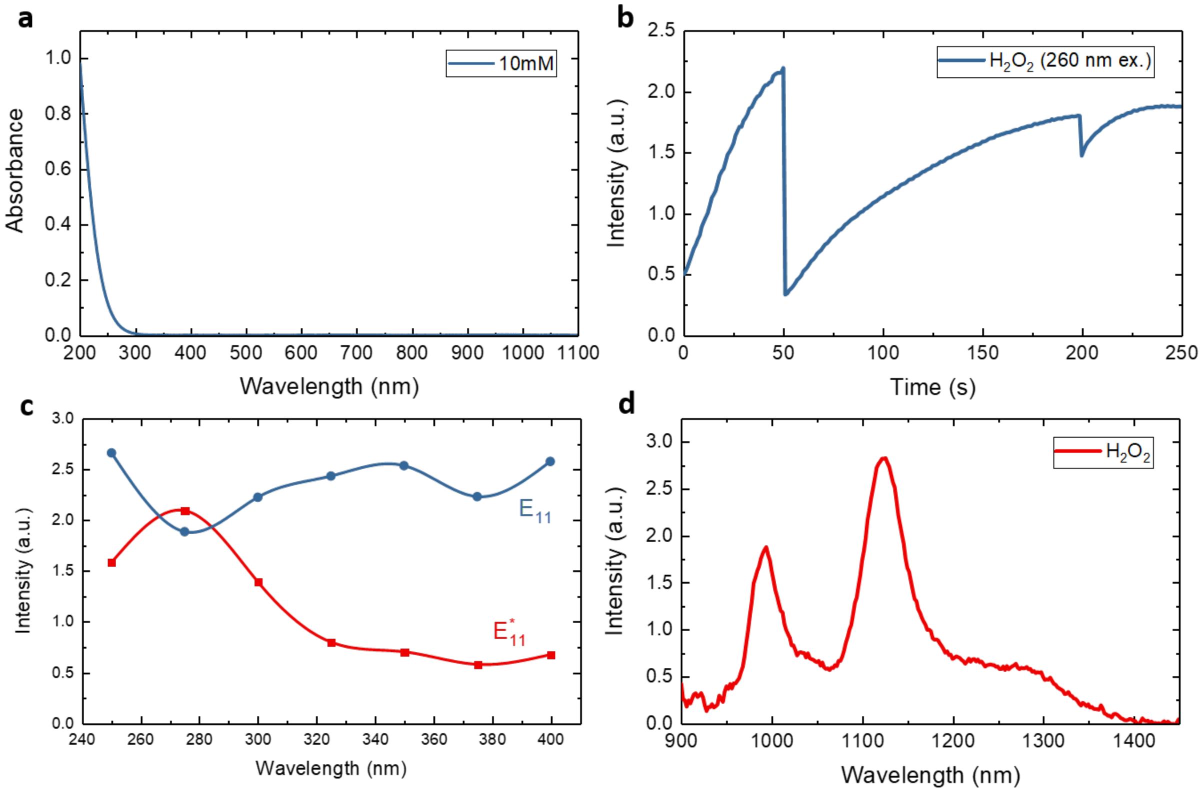

Supplementary Fig. 26 Oxygen doping of CoMoCAT SWCNTs using H_2O_2. **a**, Absorption spectrum of H_2O_2. **b**, Reaction kinetics excited at 260 nm. Three doping steps were used. **c**, Action spectra of E_{11} and E_{11}^* peaks. **d**, Fluorescence spectrum after oxygen doping.

High-throughput flow reactor. The LED irradiation light source can give a maximum power density of ~73 $mWcm^{-2}$. A quartz condenser lens was used to focus its output light into a 3 mm diameter cylindrical beam. Reactant injection rates were controlled by a dual syringe pump (Harvard PUMP 33). The SWCNTs and NaClO were mixed right before injection to prevent unwanted aggregation and side reactions. We manually added extra SC into the collection vial to cease any aggregation and side reactions. Emission spectra of the O-doped SWCNTs could be monitored *in situ*. We collected the fluorescence from a focused spot near the quartz window to reduce internal absorption when using high SWCNT concentrations.

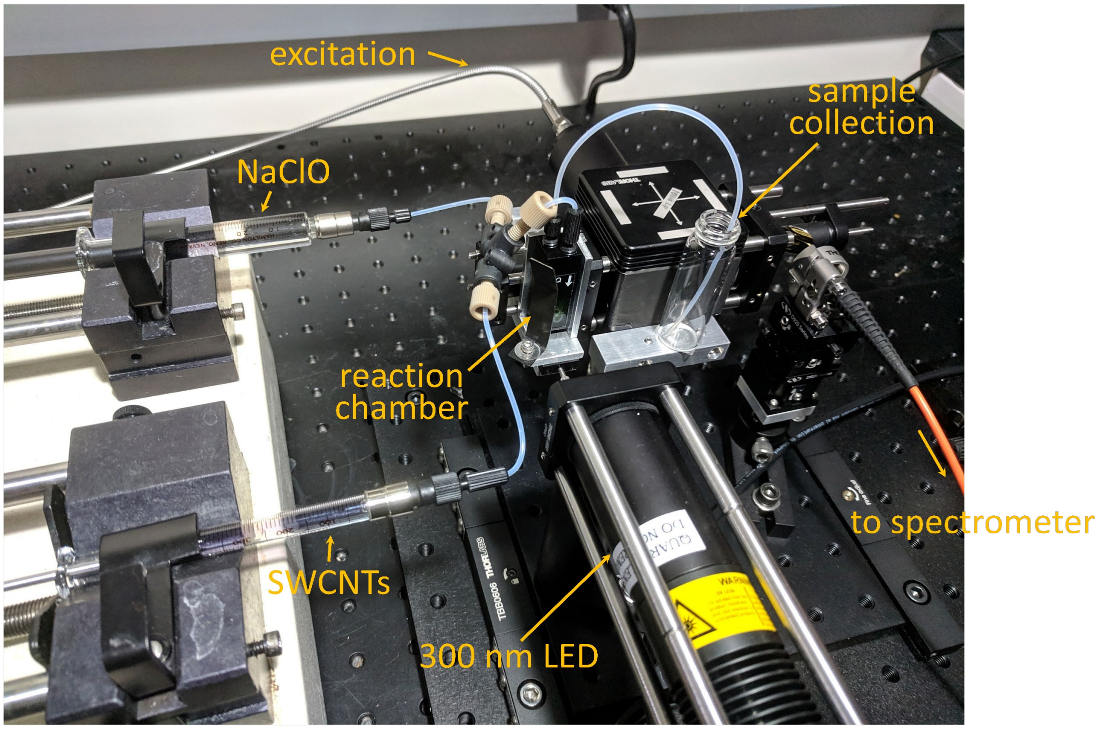

Supplementary Fig. 27 Photo of the flow reactor.

***In vivo* imaging.** The *in vivo* imaging was performed using nu/nu nude, BALB/c, or BL6 mice. About 0.7 ng μL^{-1} of DSPE-PEG_{5k} was added into as-prepared O-doped SWCNTs and the sample was dialyzed against water for 3 days. The resulting DSPE-PEG_{5k}-coated SWCNTs in 1x PBS were injected into the tail vein (~150 µL) and the image was taken starting right after the injection. The mouse was illuminated with 980 nm laser and the nanotube emission was filtered by a 1150 nm long-pass filter before imaging with the InGaAs camera. The specimen's vascular structure could be visualized clearly in the first hour after injection. To study the lymphatic drainage, ~15 µL of the same SWCNT samples were injected into the footpads and images were taken several minutes later. Supplementary Fig. 28 shows several of these SWIR images.

We note that the current standard of oncologic care relies heavily on the ability to locate sentinel nodes to cancer, followed by characterizing their shapes, sizes, uptakes, and densities. Examples of oncologic care are surgical planning, TNM model-based staging and life-span predictions, and metastatic and therapy response monitoring. Traditional modalities such as MRI, PET/CT, and ultrasound exhibit poor resolution, low reproducibility, and limited accessibility to lymph node locations. At the same time, the cost to perform these imaging modalities is usually very high. Therefore, this highly sensitive SWIR fluorescence imaging can be a potential tool to aid such traditional imaging modalities. Additionally, with the advent of immunotherapy and increased awareness of the role of the immune system in disease, better understanding and visualization of the lymphatic vessels and their cell populations are of particular relevance. Those questions could potentially be addressed using O-doped SWCNTs that are conjugated with extra targeting agents.

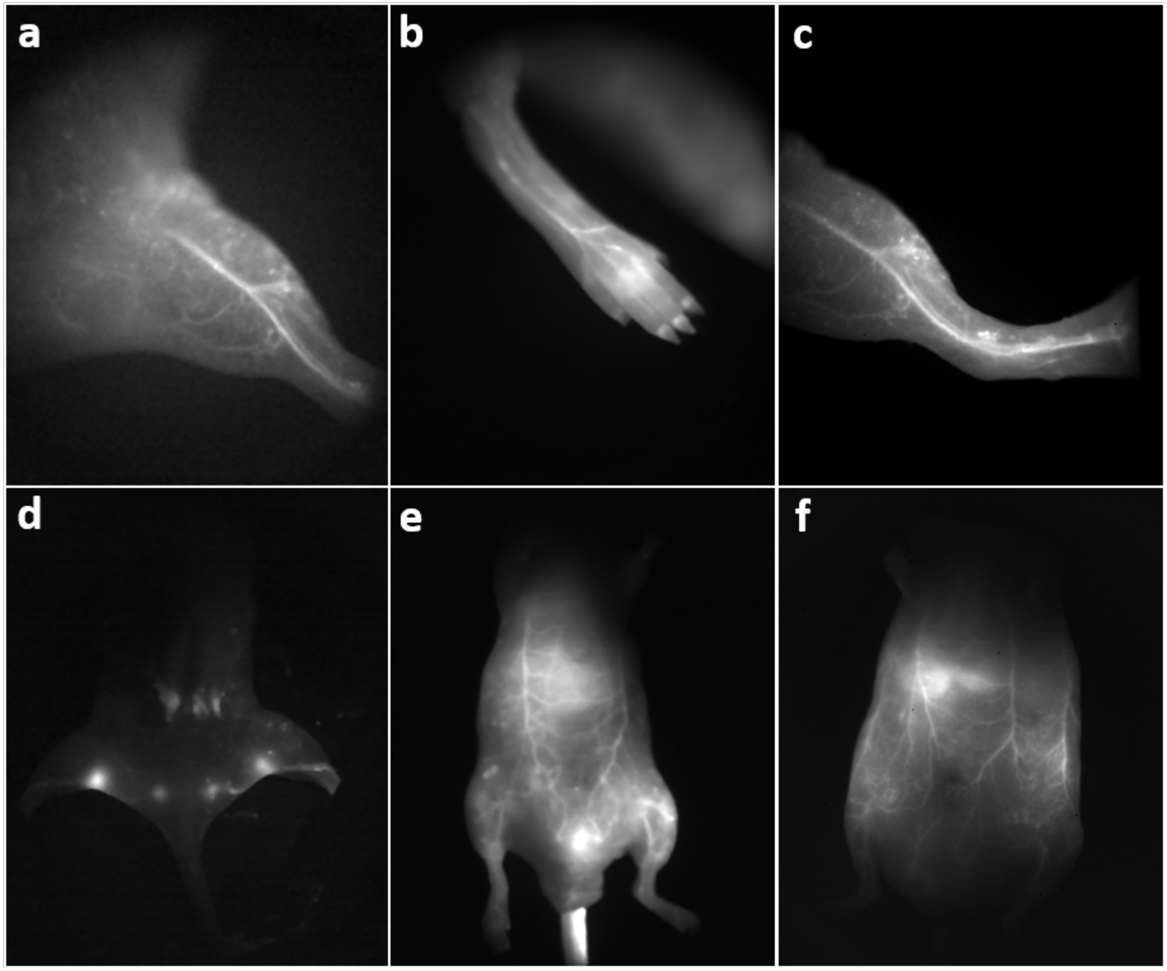

Supplementary Fig. 28 Some other *in vivo* images. **a**, Mouse leg showing femoral artery and vein. **b**, Mouse footpad **c**, Mouse leg showing medial marginal artery and vein. **d**, Lymph nodes after footpad subcutaneous injection. **e**, Whole body vasculature imaging of a nude mouse. **f**, Whole body vasculature imaging of a shaved black mouse.

Supplementary References

1. Wei, X. J., et al. High-yield and high-throughput single-chirality enantiomer separation of single-wall carbon nanotubes. *Carbon* **132**, 1-7 (2018).
2. Wei, X. J., Tanaka, T., Hirakawa, T., Wang, G. W. & Kataura, H. High-efficiency separation of (6,5) carbon nanotubes by stepwise elution gel chromatography. *Phys. Status Solidi B* **254**, 1700279 (2017).
3. Kim, M., et al. Fluorescent carbon nanotube defects manifest substantial vibrational reorganization. *J. Phys. Chem. C* **120**, 11268-11276 (2016).
4. Akizuki, N., Aota, S., Mouri, S., Matsuda, K. & Miyauchi, Y. Efficient near-infrared up-conversion photoluminescence in carbon nanotubes. *Nat. Commun.* **6**, 8920-8920 (2015).
5. Danné, N., et al. Comparative analysis of photoluminescence and upconversion emission from individual carbon nanotubes for bioimaging applications. *ACS Photonics* **5**, 359-364 (2018).
6. Magg, M., Kadria-Vili, Y., Oulevey, P., Weisman, R. B. & Buergi, T. Resonance Raman optical activity spectra of single-walled carbon nanotube enantiomers. *J. Phys. Chem. Lett.* **7**, 221-225 (2016).
7. Liu, H., Nishide, D., Tanaka, T. & Kataura, H. Large-scale single-chirality separation of single-wall carbon nanotubes by simple gel chromatography. *Nat. Commun.* **2**, 309 (2011).
8. Chiu, C. F., et al. Enzyme-catalyzed oxidation facilitates the return of fluorescence for single-walled carbon nanotubes. *J. Am. Chem. Soc.* **135**, 13356-13364 (2013).
9. Newman, L., et al. Hypochlorite degrades 2D graphene oxide sheets faster than 1D oxidised carbon nanotubes and nanohorns. *npj 2D Mater. Appl.* **1**, 39 (2017).
10. Buxton, G. V. & Subhani, M. S. Radiation-chemistry and photochemistry of oxychlorine ions. 2. Photodecomposition of aqueous-solutions of hypochlorite ions. *J. Chem. Soc. Faraday Trans.* **68**, 958-969 (1972).
11. Ghosh, S., Bachilo, S. M., Simonette, R. A., Beckingham, K. M. & Weisman, R. B. Oxygen doping modifies near-infrared band gaps in fluorescent single-walled carbon nanotubes. *Science* **330**, 1656-1659 (2010).
12. Rao, B., et al. Perchlorate production by photodecomposition of aqueous chlorine solutions. *Environ. Sci. Technol.* **46**, 11635-11643 (2012).
13. Lim, M. H., Gnanakaran, S. & Hochstrasser, R. M. Charge shifting in the ultrafast photoreactions of ClO^- in water. *J. Chem. Phys.* **106**, 3485-3493 (1997).
14. Kafle, T. R., et al. Hot exciton relaxation and exciton trapping in single-walled carbon nanotube thin films. *J. Phys. Chem. C* **120**, 24482-24490 (2016).
15. Slanger, T. G. & Copeland, R. A. Energetic oxygen in the upper atmosphere and the laboratory. *Chem. Rev.* **103**, 4731-4766 (2003).
16. Benedikt, J., et al. The fate of plasma-generated oxygen atoms in aqueous solutions: non-equilibrium atmospheric pressure plasmas as an efficient source of atomic $O_{(aq)}$. *Phys. Chem. Chem. Phys.* **20**, 12037-12042 (2018).
17. Verlackt, C. C. W., Neyts, E. C. & Bogaerts, A. Atomic scale behavior of oxygen-based radicals in water. *J. Phys. D: Appl. Phys.* **50**, 11LT01 (2017).
18. Codorniu-Hernández, E., Hall, K. W., Ziemianowicz, D., Carpendale, S. & Kusalik, P. G. Aqueous production of oxygen atoms from hydroxyl radicals. *Phys. Chem. Chem. Phys.* **16**, 26094-26102 (2014).

19. Feng, Y. G., Smith, D. W. & Bolton, J. R. Photolysis of aqueous free chlorine species (NOCl and OCl^-) with 254 nm ultraviolet light. *J. Environ. Eng. Sci.* **6**, 277-284 (2007).
20. Streit, J. K., Bachilo, S. M., Sanchez, S. R., Lin, C.-W. & Weisman, R. B. Variance spectroscopy. *J. Phys. Chem. Lett.* **6**, 3976-3981 (2015).
21. Chiu, C. F., Saidi, W. A., Kagan, V. E. & Star, A. Defect-induced near-infrared photoluminescence of single-walled carbon nanotubes treated with polyunsaturated fatty acids. *J. Am. Chem. Soc.* **139**, 4859-4865 (2017).
22. Rao, A. S. Photodecomposition and absorption spectrum of potassium permanganate. *Proc. Indian Acad. Sci. A* **6**, 293-300 (1937).
23. Houmoller, J., et al. On the photoabsorption by permanganate ions in vacuo and the role of a single water molecule. New experimental benchmarks for electronic structure theory. *ChemPhysChem* **14**, 1133-1137 (2013).
24. Hunt, J. P. & Taube, H. The photochemical decomposition of hydrogen peroxide. Quantum yields, tracer and fractionation effects. *J. Am. Chem. Soc.* **74**, 5999-6002 (1952).
